# Supplementary material for: Glutamylation imbalance impairs the molecular architecture of the photoreceptor cilium
Source: EMBO J. 2024 Nov 11;43(24):19. doi: 10.1038/s44318-024-00284-1 (PMC11649768; doi:10.1038/s44318-024-00284-1)
Supplement: Supplementary file 1 — Appendix [file 44318_2024_284_MOESM1_ESM.pdf]

Appendix

Appendix Figure S1: Glycylation signal at the level of the photoreceptor basal body

Appendix Figure S2: Polyglutamylation signal enrichment at the ciliary base

Appendix Figure S3: Delta2 tubulin signal in the OS

Appendix Figure S4: Connecting cilium inner scaffold in human photoreceptor cells

Appendix Figure S5: Loss of acetylation in the OS in *Atat1*$^{-/-}$ mice

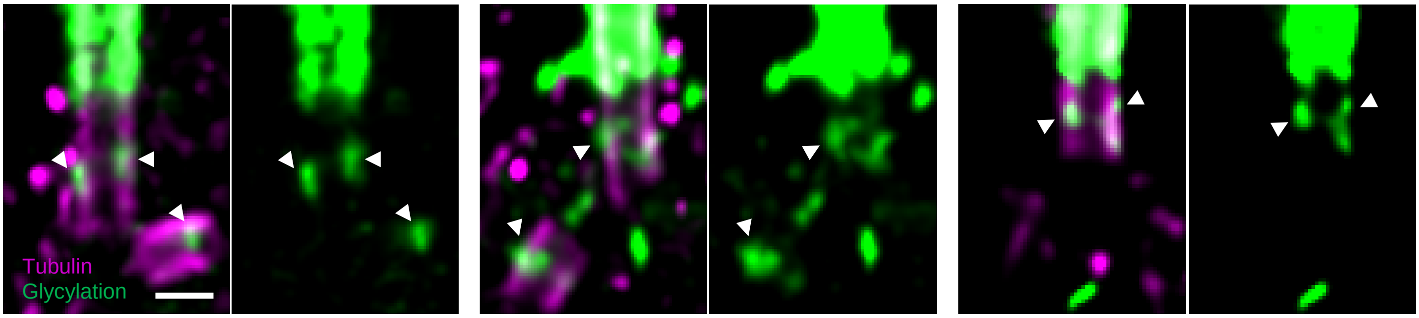

Appendix Figure S1: Glycylation signal at the level of the photoreceptor basal body

Expanded photoreceptor cell centrioles stained for TAP952 (green, saturated) and tubulin (magenta). Glycylation is observed at the level of centriole subdistal appendages (white arrowheads) when the TAP952 signal is pushed. Scale bar: 200 nm.

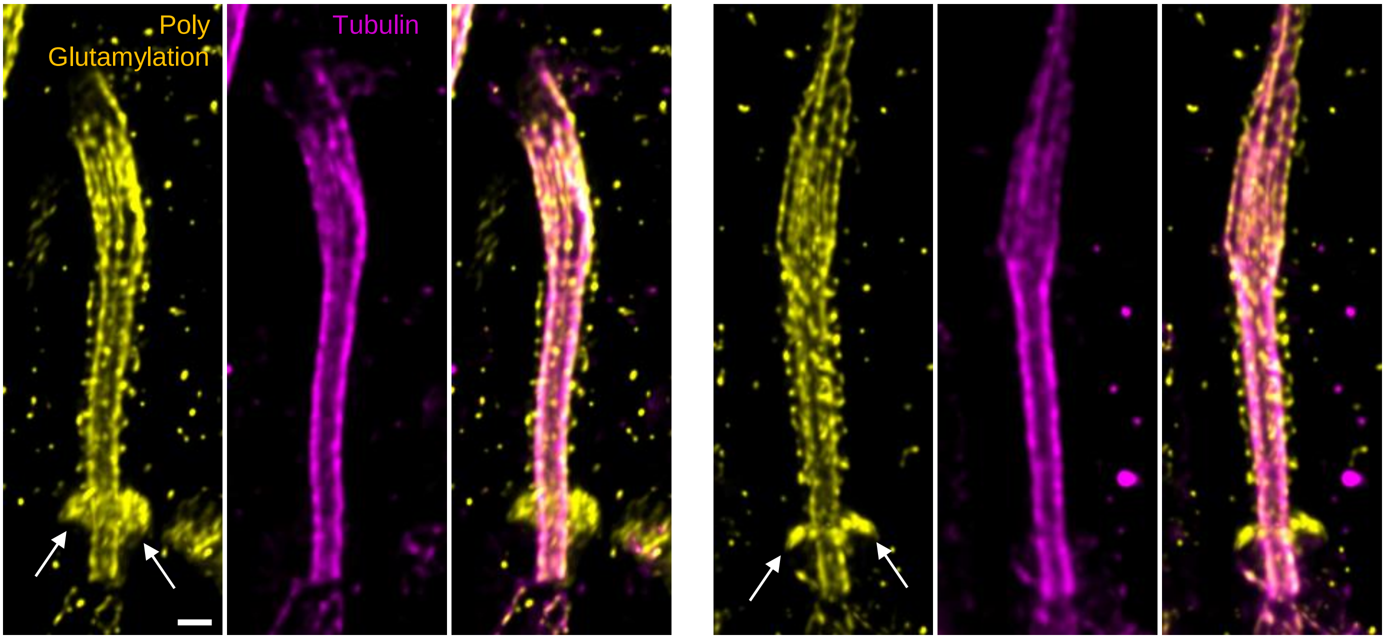

Appendix Figure S2: Polyglutamylation signal enrichment at the ciliary base

Expanded photoreceptor cell outer segments stained with PolyE (yellow) and tubulin (magenta). Polyglutamylation is sometimes enriched at the base of the cilium, similar to IFT trains formation. Scale bar: 200 nm.

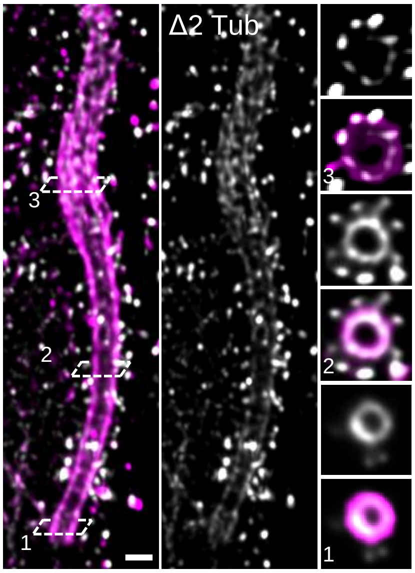

Appendix Figure S3: Delta2 tubulin signal in the OS

Molecular mapping of the delta2 tubulin (gray) along the photoreceptor outer segment axoneme stained for tubulin (magenta). Transversal section images corresponding to different regions of the OS (centriole (1), connecting cilium (2) and bulge (3), depicted by the dashed lines and numbers on longitudinal images) are represented on the right side. Scalebar: 200 nm.

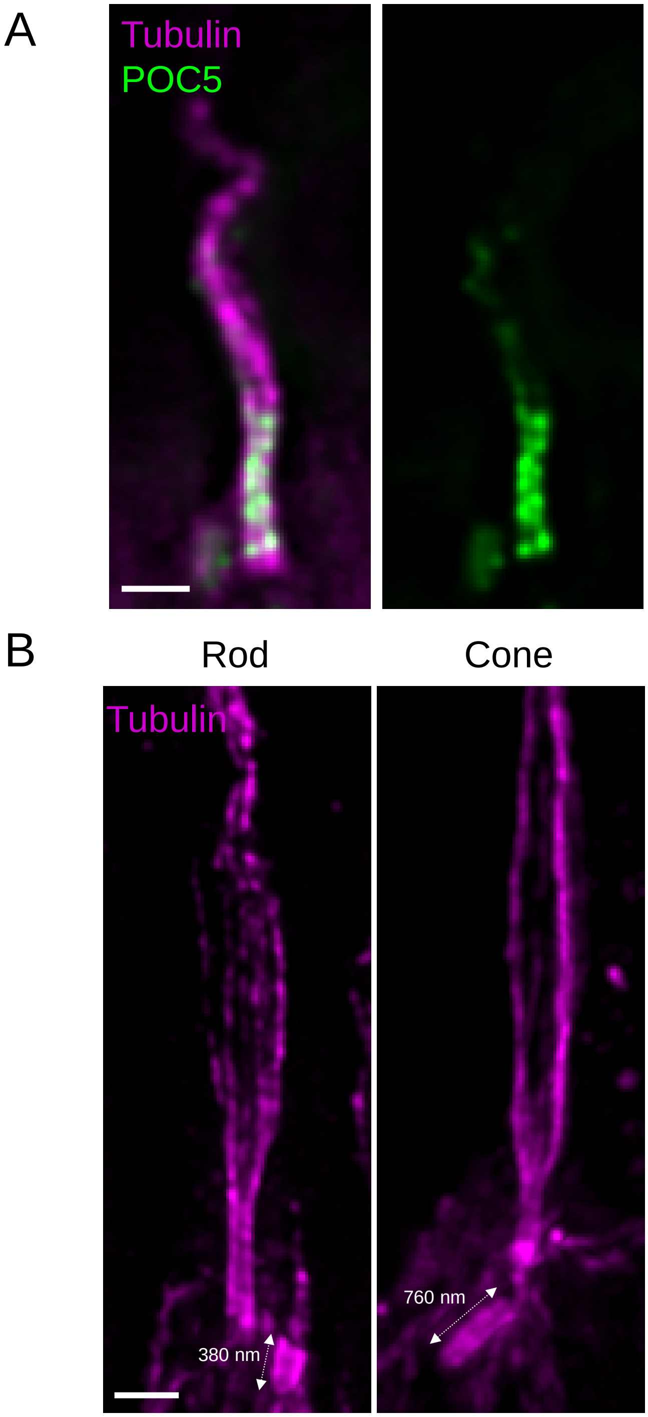

Appendix Figure S4: Connecting cilium inner scaffold in human photoreceptor cells

(**A**) Expanded human rod stained for POC5 (green) and tubulin (magenta). (**B**) Expanded human rod (left) or cone (right) photoreceptor cells stained for tubulin (magenta). Size of the daughter centrioles are highlighted by the white double headed arrows. Scale bar: 500 nm.

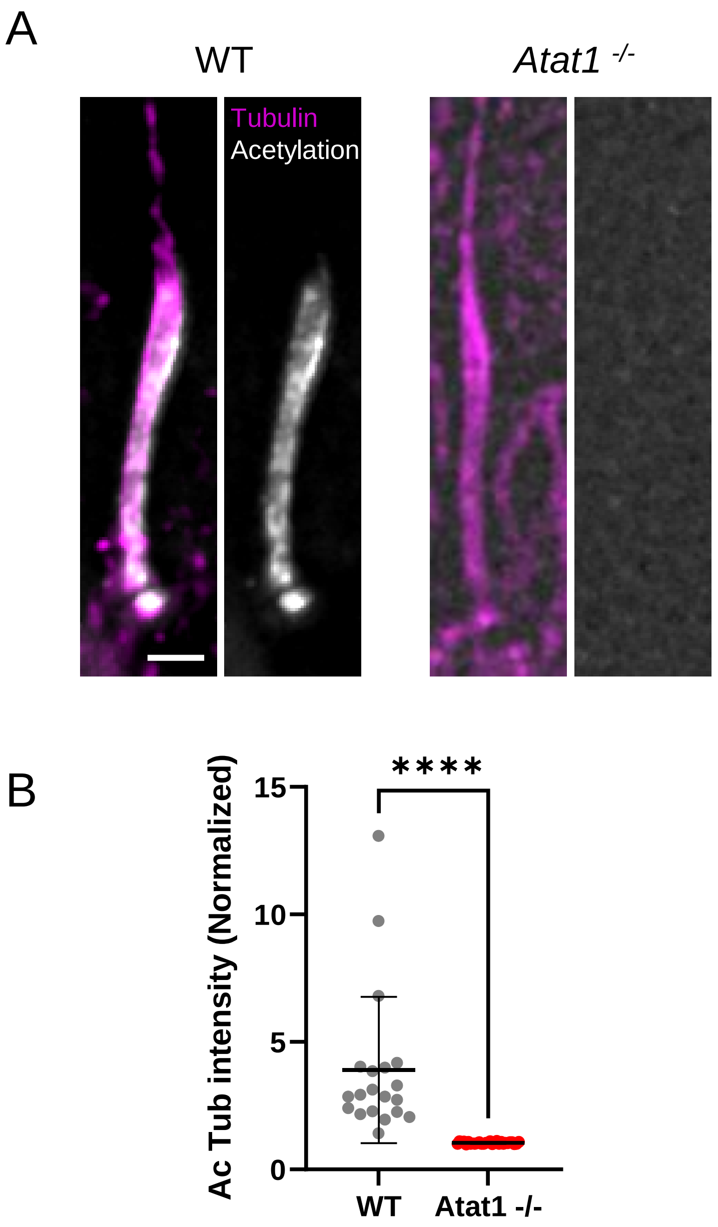

Appendix Figure S5: Loss of acetylation in the OS in *Atat1$^{-/-}$* mice

(**A**) Expanded WT or *Atat1$^{-/-}$* photoreceptor cells stained for acetylated tubulin (gray), together with tubulin (magenta) demonstrating the total loss of acetylation in *Atat1$^{-/-}$*. Scalebar: 500 nm. (**B**) Quantification of the acetylated tubulin signal in WT or *Atat1$^{-/-}$* photoreceptor cells. WT: 3.9 +/- 2.9; *Atat1$^{-/-}$*: 1.04 +/- 0.04 (Mean +/- SD). N=3 animals. Test: Two-tailed Mann-Whitney test. WT vs. Atat1$^{-/-}$: **** (Adjusted P value: <0.0001).

Appendix Figure S5
