# Supplementary figures and images for: Glutamylation imbalance impairs the molecular architecture of the photoreceptor cilium (part 1 of 2)
Source: EMBO J. 2024 Nov 11;43(24):19. doi: 10.1038/s44318-024-00284-1 (PMC11649768; doi:10.1038/s44318-024-00284-1)

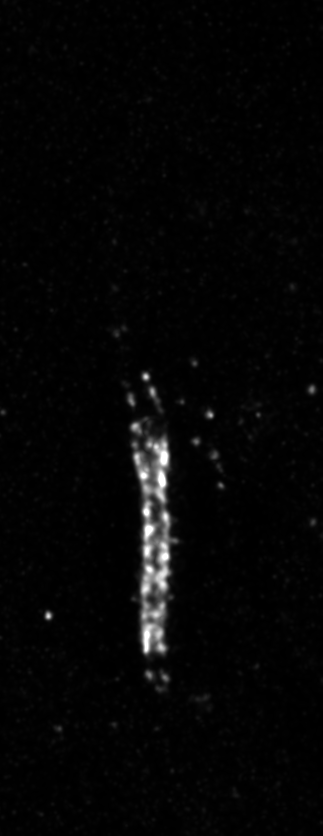

Supplement: Supplementary file 3 — Source data Fig. 1 [file 44318_2024_284_MOESM3_ESM.zip › EMBOJ-2024-118613-T _SourceDataForFigure1/1A/2.tif]

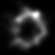

Supplement: Supplementary file 3 — Source data Fig. 1 [file 44318_2024_284_MOESM3_ESM.zip › EMBOJ-2024-118613-T _SourceDataForFigure1/1A/bulge-1 colored sized.tif]

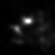

Supplement: Supplementary file 3 — Source data Fig. 1 [file 44318_2024_284_MOESM3_ESM.zip › EMBOJ-2024-118613-T _SourceDataForFigure1/1A/centriole-crop.tif]

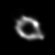

Supplement: Supplementary file 3 — Source data Fig. 1 [file 44318_2024_284_MOESM3_ESM.zip › EMBOJ-2024-118613-T _SourceDataForFigure1/1A/TAP Top view.tif]

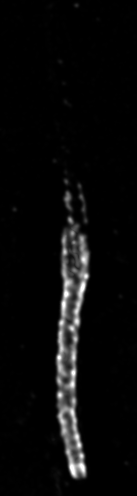

Supplement: Supplementary file 3 — Source data Fig. 1 [file 44318_2024_284_MOESM3_ESM.zip › EMBOJ-2024-118613-T _SourceDataForFigure1/1B/ac tb 2.tif]

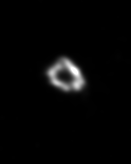

Supplement: Supplementary file 3 — Source data Fig. 1 [file 44318_2024_284_MOESM3_ESM.zip › EMBOJ-2024-118613-T _SourceDataForFigure1/1B/bulge.tif]

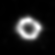

Supplement: Supplementary file 3 — Source data Fig. 1 [file 44318_2024_284_MOESM3_ESM.zip › EMBOJ-2024-118613-T _SourceDataForFigure1/1B/CC 13-1-1.tif]

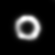

Supplement: Supplementary file 3 — Source data Fig. 1 [file 44318_2024_284_MOESM3_ESM.zip › EMBOJ-2024-118613-T _SourceDataForFigure1/1B/centriole-1.tif]

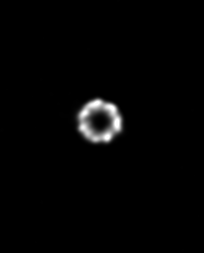

Supplement: Supplementary file 3 — Source data Fig. 1 [file 44318_2024_284_MOESM3_ESM.zip › EMBOJ-2024-118613-T _SourceDataForFigure1/1C/GT CC 5.tif]

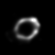

Supplement: Supplementary file 3 — Source data Fig. 1 [file 44318_2024_284_MOESM3_ESM.zip › EMBOJ-2024-118613-T _SourceDataForFigure1/1C/GT top view centriole.tif]

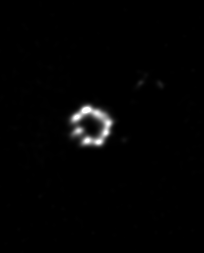

Supplement: Supplementary file 3 — Source data Fig. 1 [file 44318_2024_284_MOESM3_ESM.zip › EMBOJ-2024-118613-T _SourceDataForFigure1/1C/GT335 bulge 2.tif]

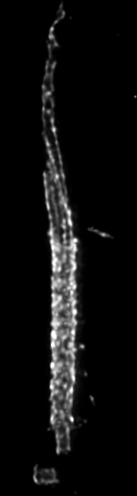

Supplement: Supplementary file 3 — Source data Fig. 1 [file 44318_2024_284_MOESM3_ESM.zip › EMBOJ-2024-118613-T _SourceDataForFigure1/1C/GT335.tif]

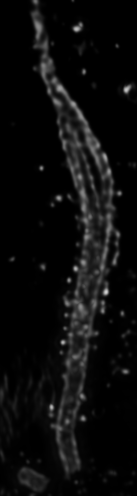

Supplement: Supplementary file 3 — Source data Fig. 1 [file 44318_2024_284_MOESM3_ESM.zip › EMBOJ-2024-118613-T _SourceDataForFigure1/1D/3-1.tif]

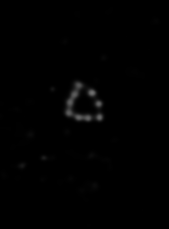

Supplement: Supplementary file 3 — Source data Fig. 1 [file 44318_2024_284_MOESM3_ESM.zip › EMBOJ-2024-118613-T _SourceDataForFigure1/1D/polyE bulge 2.tif]

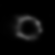

Supplement: Supplementary file 3 — Source data Fig. 1 [file 44318_2024_284_MOESM3_ESM.zip › EMBOJ-2024-118613-T _SourceDataForFigure1/1D/polyE centriole-1 sized.tif]

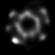

Supplement: Supplementary file 3 — Source data Fig. 1 [file 44318_2024_284_MOESM3_ESM.zip › EMBOJ-2024-118613-T _SourceDataForFigure1/1D/polyE top view CC.tif]

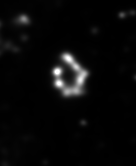

Supplement: Supplementary file 3 — Source data Fig. 1 [file 44318_2024_284_MOESM3_ESM.zip › EMBOJ-2024-118613-T _SourceDataForFigure1/1E/bulge.tif]

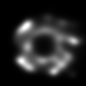

Supplement: Supplementary file 3 — Source data Fig. 1 [file 44318_2024_284_MOESM3_ESM.zip › EMBOJ-2024-118613-T _SourceDataForFigure1/1E/detyr tub top CC.tif]

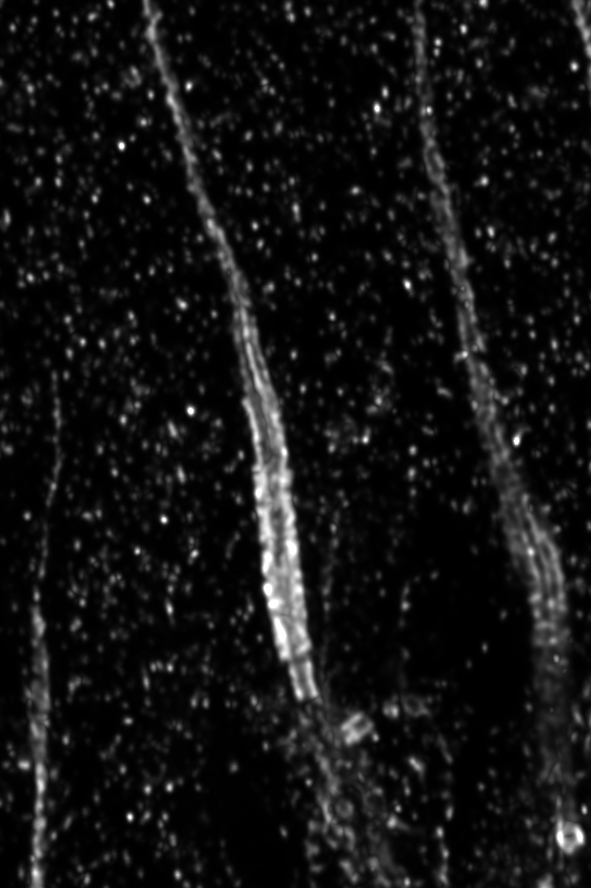

Supplement: Supplementary file 3 — Source data Fig. 1 [file 44318_2024_284_MOESM3_ESM.zip › EMBOJ-2024-118613-T _SourceDataForFigure1/1E/side.tif]

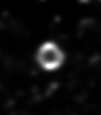

Supplement: Supplementary file 3 — Source data Fig. 1 [file 44318_2024_284_MOESM3_ESM.zip › EMBOJ-2024-118613-T _SourceDataForFigure1/1E/top centriole.tif]

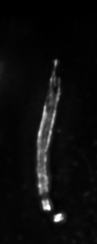

Supplement: Supplementary file 3 — Source data Fig. 1 [file 44318_2024_284_MOESM3_ESM.zip › EMBOJ-2024-118613-T _SourceDataForFigure1/1G/MAX_P10 het ni 2 gt335 tub.lif - Series004_Lng_SVCC-1-1.tif]

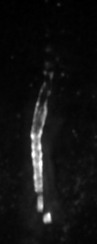

Supplement: Supplementary file 3 — Source data Fig. 1 [file 44318_2024_284_MOESM3_ESM.zip › EMBOJ-2024-118613-T _SourceDataForFigure1/1G/MAX_P14 HET NI 1 GT335 tub.lif - Series002_Lng_SVCC-1-1.tif]

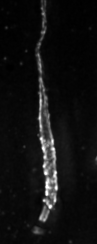

Supplement: Supplementary file 3 — Source data Fig. 1 [file 44318_2024_284_MOESM3_ESM.zip › EMBOJ-2024-118613-T _SourceDataForFigure1/1G/MAX_P22 HET NI 2 GT335 tub.lif - Series001_Lng_SVCC-1-1.tif]

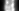

Supplement: Supplementary file 3 — Source data Fig. 1 [file 44318_2024_284_MOESM3_ESM.zip › EMBOJ-2024-118613-T _SourceDataForFigure1/1H/MAX_P10 het ni 2 gt335 tub.lif - Series004_Lng_SVCC-1-1-1.tif]

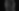

Supplement: Supplementary file 3 — Source data Fig. 1 [file 44318_2024_284_MOESM3_ESM.zip › EMBOJ-2024-118613-T _SourceDataForFigure1/1H/MAX_P14 HET NI 1 GT335 tub.lif - Series002_Lng_SVCC-1-1-1.tif]

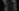

Supplement: Supplementary file 3 — Source data Fig. 1 [file 44318_2024_284_MOESM3_ESM.zip › EMBOJ-2024-118613-T _SourceDataForFigure1/1H/MAX_P22 HET NI 2 GT335 tub.lif - Series001_Lng_SVCC-1-1-1.tif]

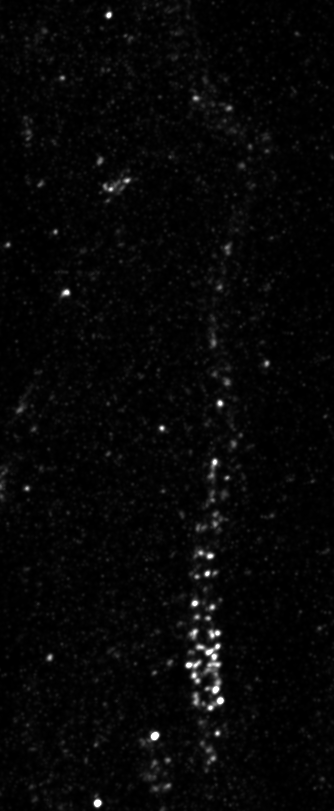

Supplement: Supplementary file 4 — Source data Fig. 2 [file 44318_2024_284_MOESM4_ESM.zip › EMBOJ-2024-118613-T _SourceDataForFigure2/2A/1-1.tif]

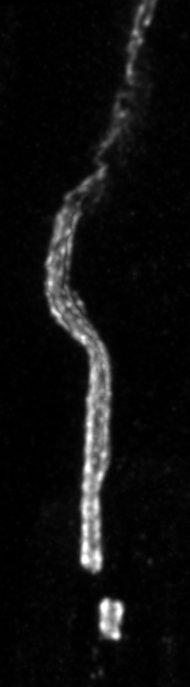

Supplement: Supplementary file 4 — Source data Fig. 2 [file 44318_2024_284_MOESM4_ESM.zip › EMBOJ-2024-118613-T _SourceDataForFigure2/2B/1-1.tif]

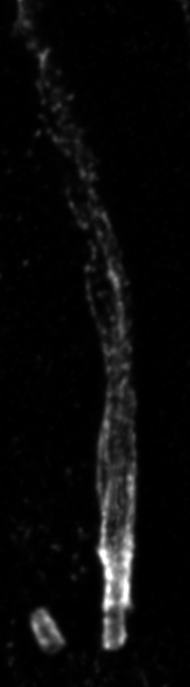

Supplement: Supplementary file 4 — Source data Fig. 2 [file 44318_2024_284_MOESM4_ESM.zip › EMBOJ-2024-118613-T _SourceDataForFigure2/2C/2-1.tif]

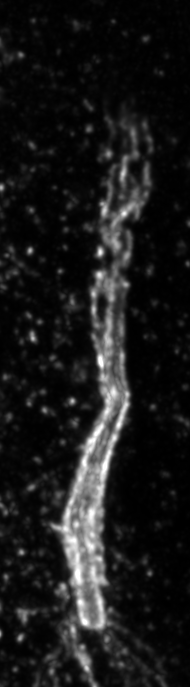

Supplement: Supplementary file 4 — Source data Fig. 2 [file 44318_2024_284_MOESM4_ESM.zip › EMBOJ-2024-118613-T _SourceDataForFigure2/2D/1-1.tif]

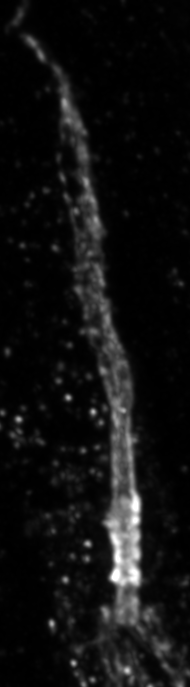

Supplement: Supplementary file 4 — Source data Fig. 2 [file 44318_2024_284_MOESM4_ESM.zip › EMBOJ-2024-118613-T _SourceDataForFigure2/2E/4-1.tif]

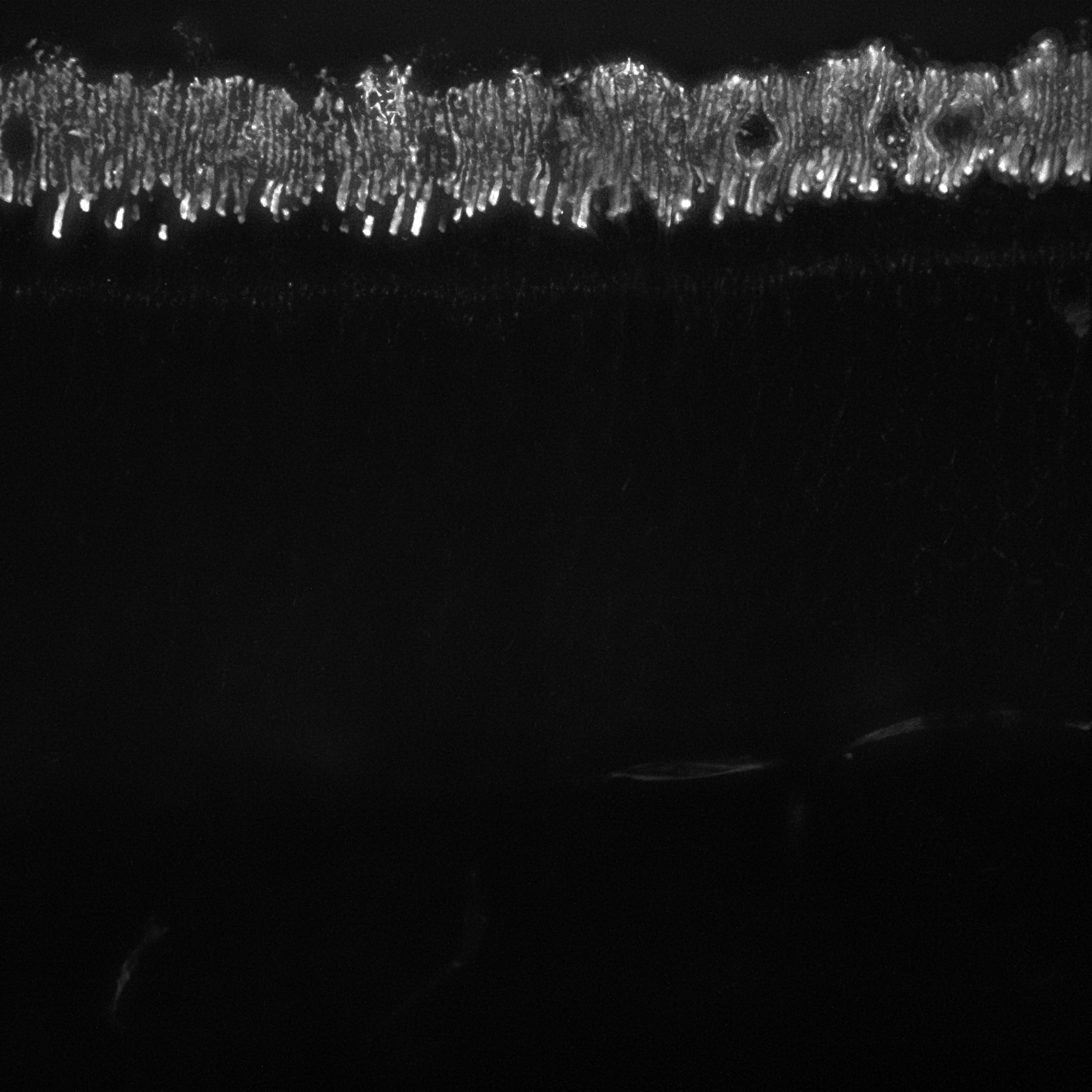

Supplement: Supplementary file 5 — Source data Fig. 3 [file 44318_2024_284_MOESM5_ESM.zip › EMBOJ-2024-118613-T _SourceDataForFigure3/3A/MAX_ATAT WT 91 18M rhodo tub.lif - Series002_Lng_SVCC.tif]

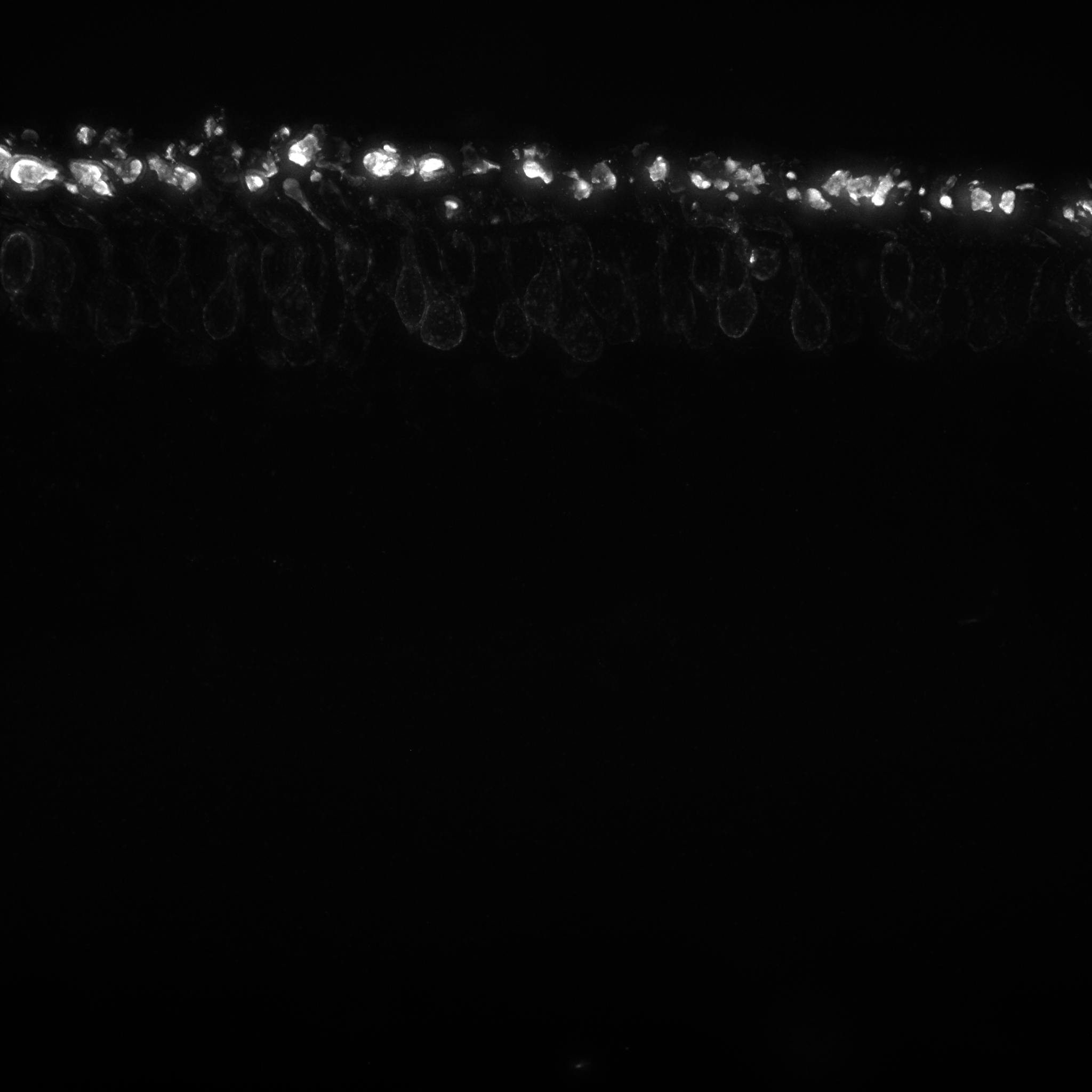

Supplement: Supplementary file 5 — Source data Fig. 3 [file 44318_2024_284_MOESM5_ESM.zip › EMBOJ-2024-118613-T _SourceDataForFigure3/3B/MAX_CCP5 KO 182 rhodo tub.lif - Series004_Lng_SVCC.tif]

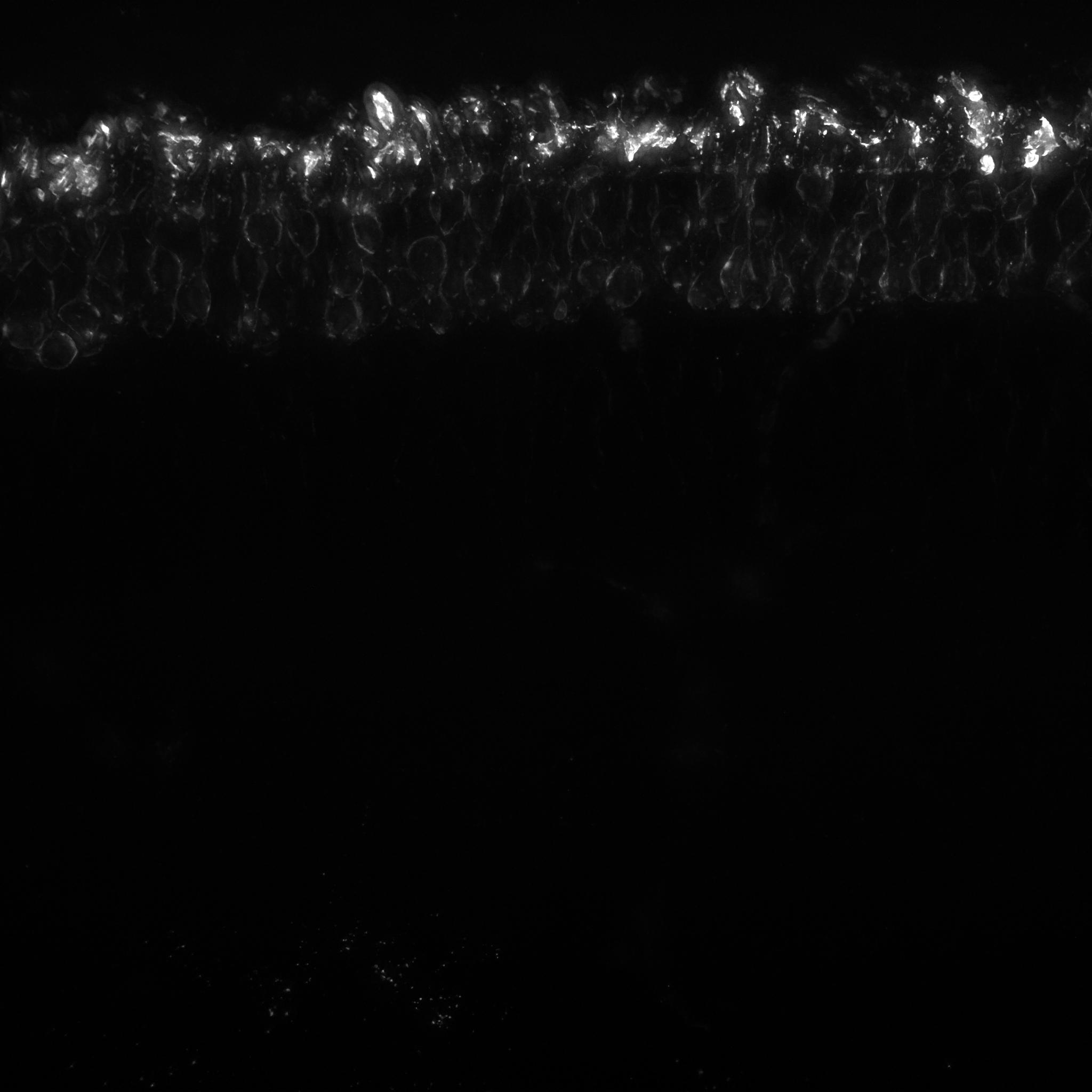

Supplement: Supplementary file 5 — Source data Fig. 3 [file 44318_2024_284_MOESM5_ESM.zip › EMBOJ-2024-118613-T _SourceDataForFigure3/3C/MAX_CCP1 ko 1180 rhodo tub.lif - Series002_Lng_SVCC.tif]

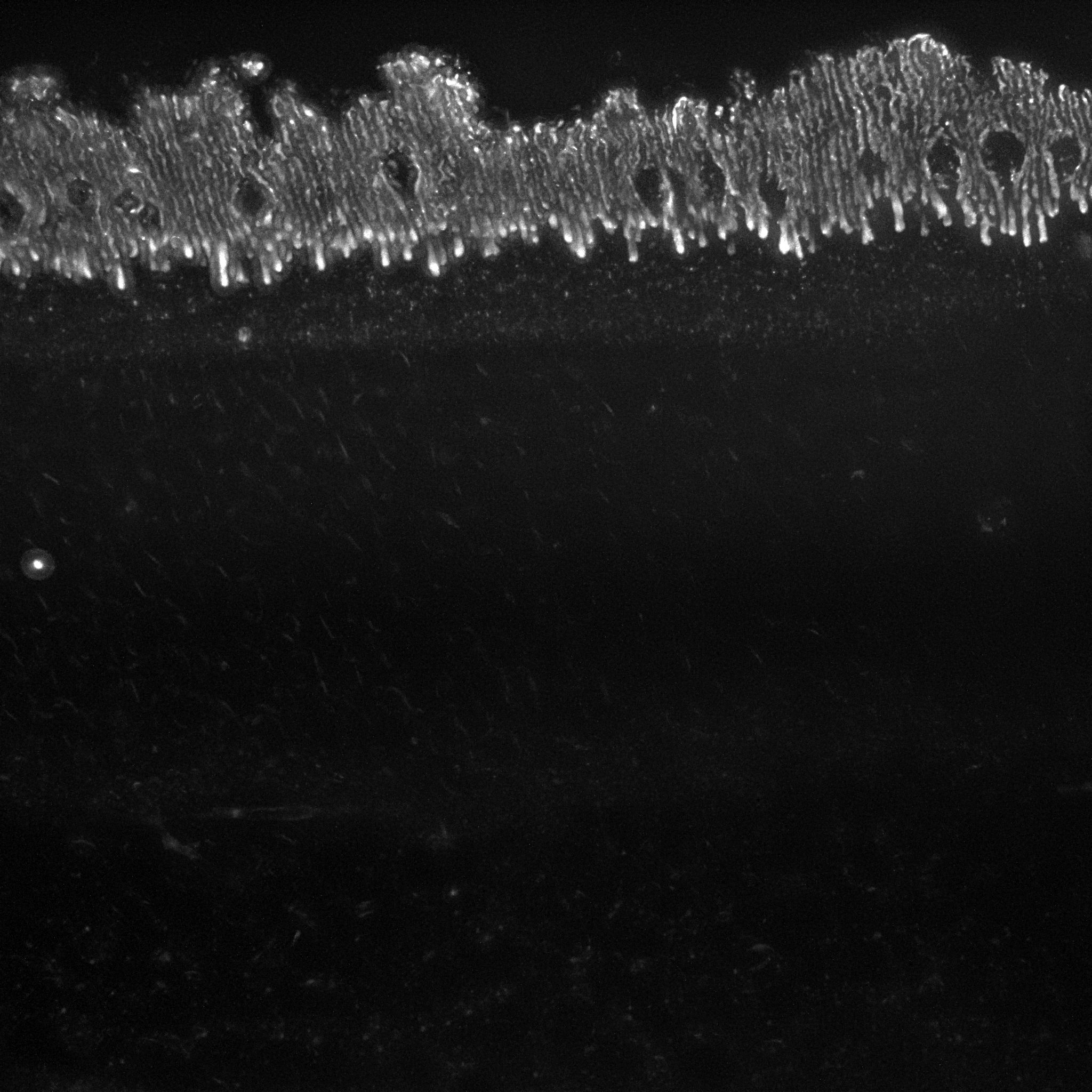

Supplement: Supplementary file 5 — Source data Fig. 3 [file 44318_2024_284_MOESM5_ESM.zip › EMBOJ-2024-118613-T _SourceDataForFigure3/3D/MAX_ATAT KO 256 18M rhodo tub .lif - Series002_Lng_SVCC.tif]

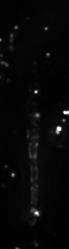

Supplement: Supplementary file 5 — Source data Fig. 3 [file 44318_2024_284_MOESM5_ESM.zip › EMBOJ-2024-118613-T _SourceDataForFigure3/3F/MAX_CCP1 WT 8M TAP952 tub.lif - Series002_Lng_SVCC-1-1-1.tif]

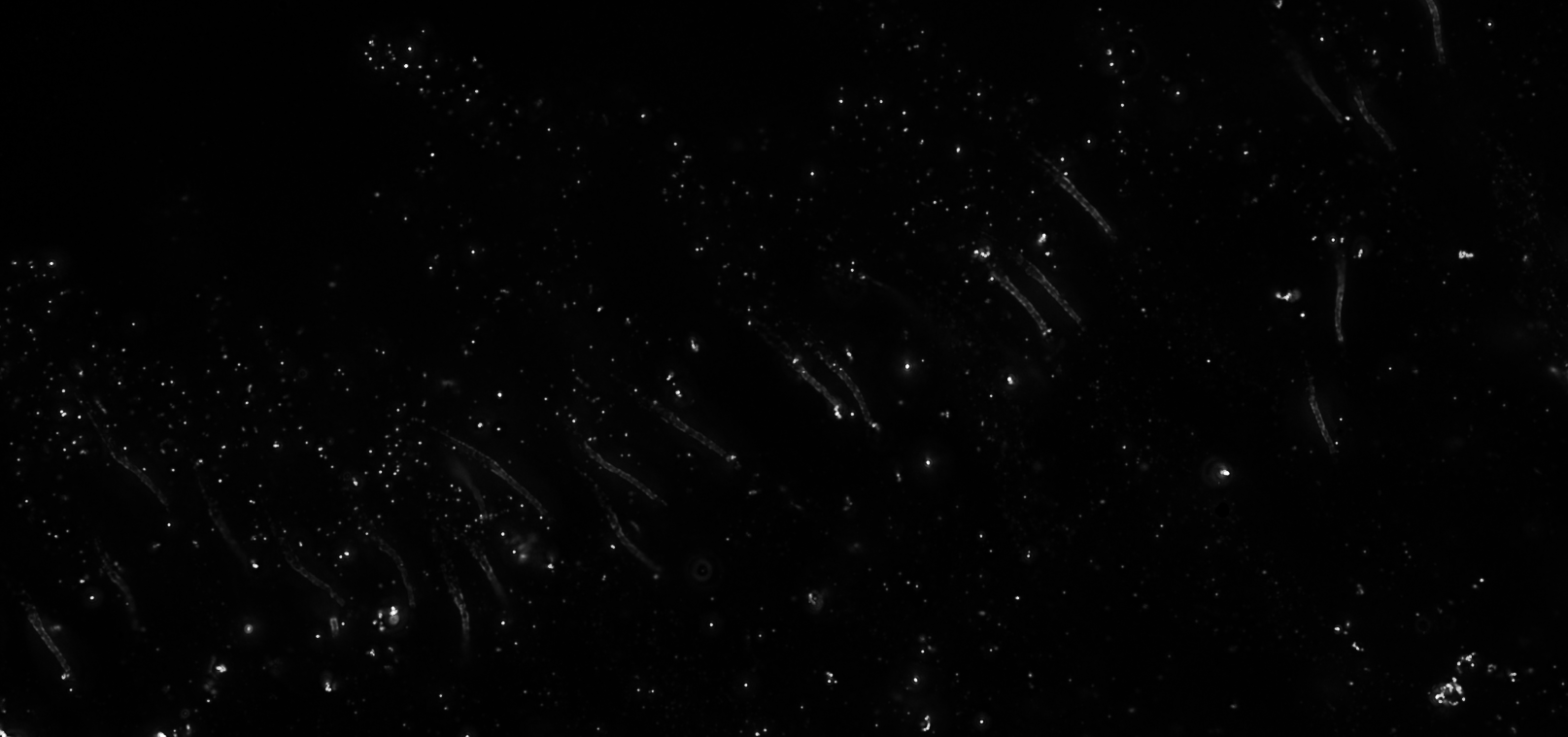

Supplement: Supplementary file 5 — Source data Fig. 3 [file 44318_2024_284_MOESM5_ESM.zip › EMBOJ-2024-118613-T _SourceDataForFigure3/3F/MAX_CCP1 WT 8M TAP952 tub.lif - Series002_Lng_SVCC-1.tif]

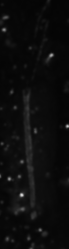

Supplement: Supplementary file 5 — Source data Fig. 3 [file 44318_2024_284_MOESM5_ESM.zip › EMBOJ-2024-118613-T _SourceDataForFigure3/3G/MAX_20220929 CCP5 KO 12M poc5tub .lif - Series006_Lng_SVCC-1-1-1.tif]

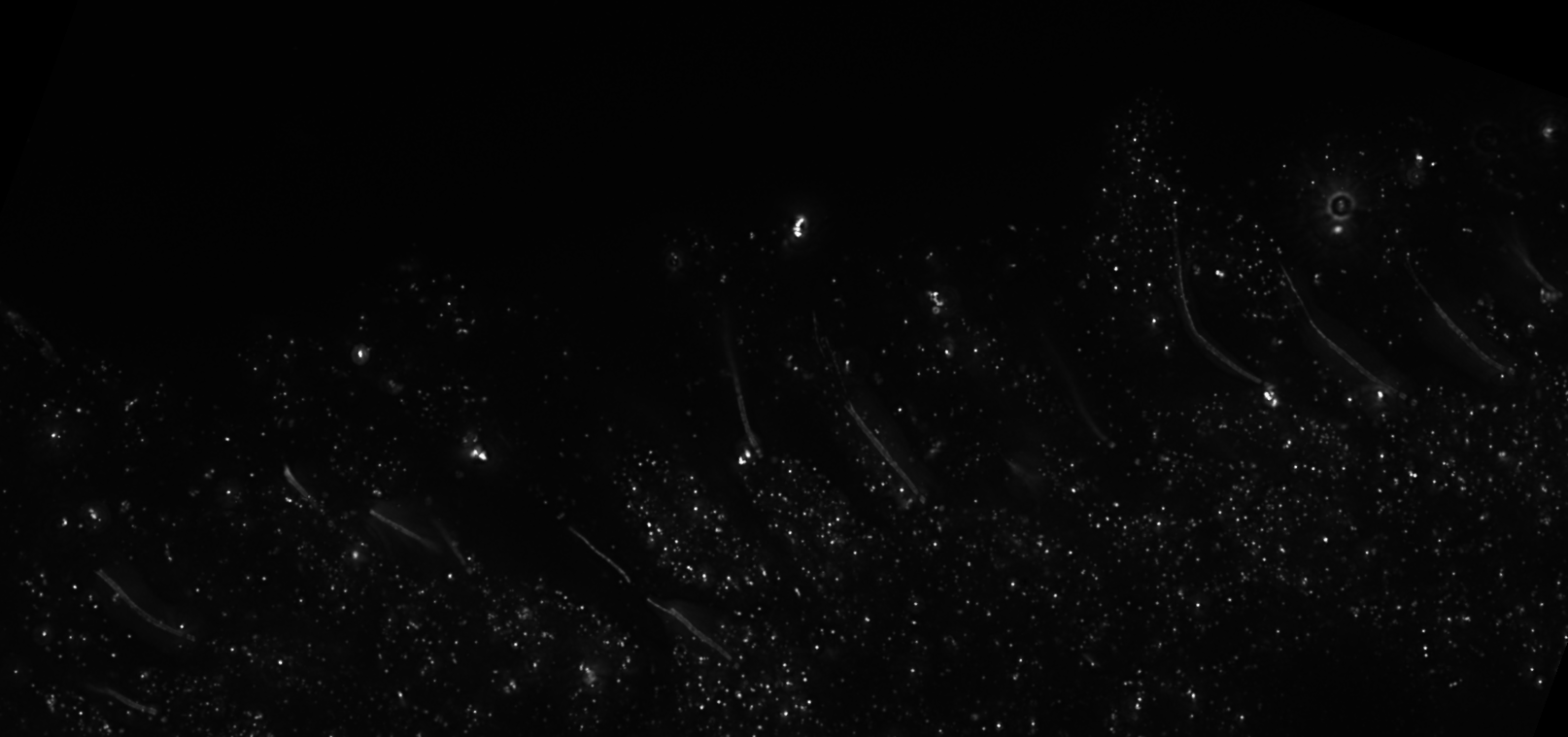

Supplement: Supplementary file 5 — Source data Fig. 3 [file 44318_2024_284_MOESM5_ESM.zip › EMBOJ-2024-118613-T _SourceDataForFigure3/3G/MAX_20220929 CCP5 KO 12M poc5tub .lif - Series006_Lng_SVCC-1.tif]

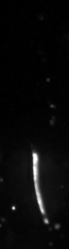

Supplement: Supplementary file 5 — Source data Fig. 3 [file 44318_2024_284_MOESM5_ESM.zip › EMBOJ-2024-118613-T _SourceDataForFigure3/3H/MAX_CCP1 KO 1180 POC5 tub .lif - Series003_Lng_SVCC-1-1.tif]

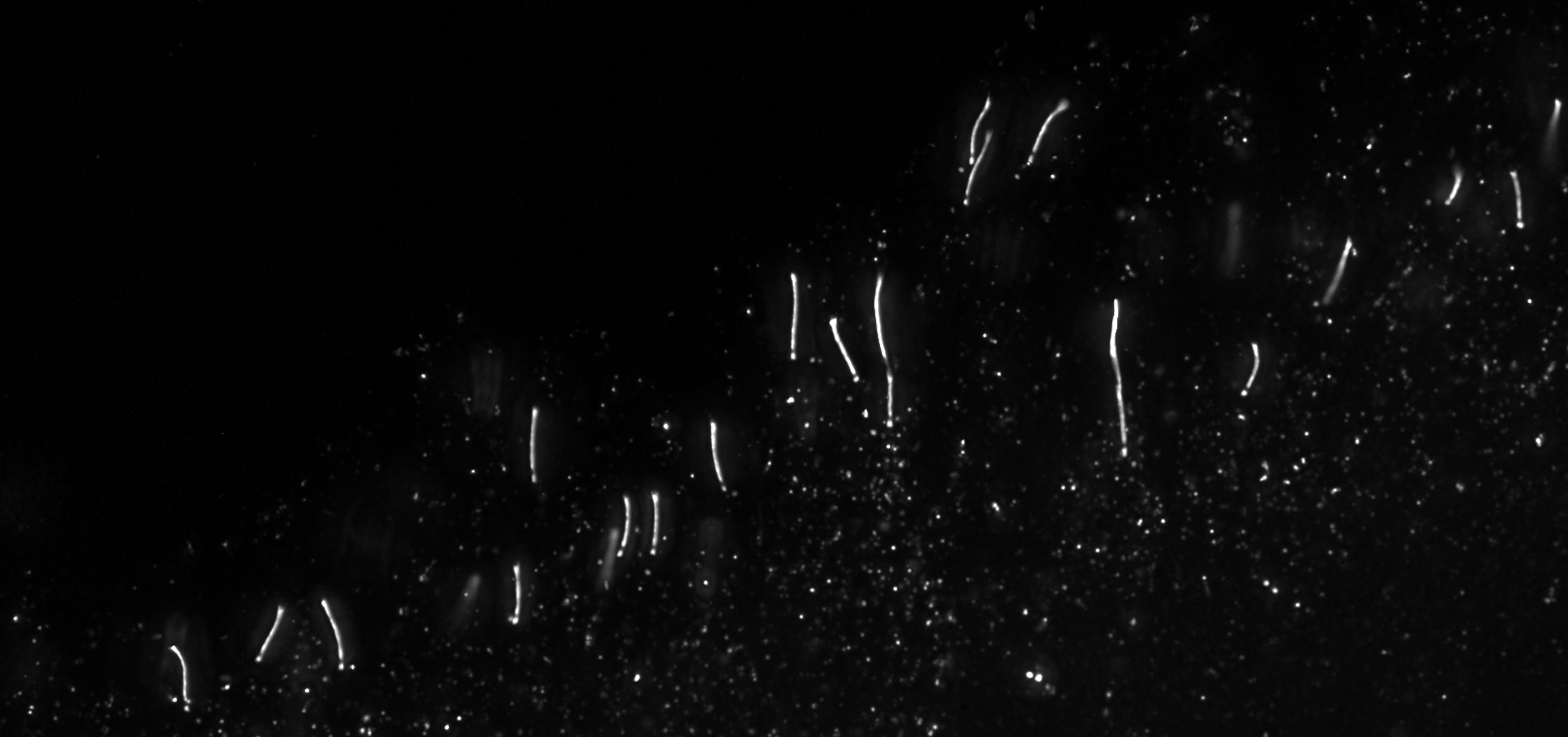

Supplement: Supplementary file 5 — Source data Fig. 3 [file 44318_2024_284_MOESM5_ESM.zip › EMBOJ-2024-118613-T _SourceDataForFigure3/3H/MAX_CCP1 KO 1180 POC5 tub .lif - Series003_Lng_SVCC-1.tif]

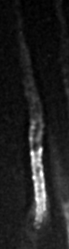

Supplement: Supplementary file 5 — Source data Fig. 3 [file 44318_2024_284_MOESM5_ESM.zip › EMBOJ-2024-118613-T _SourceDataForFigure3/3I/MAX_ATAT KO 255 GT335 TUB GP.lif - Series002_Lng_SVCC-2-1.tif]

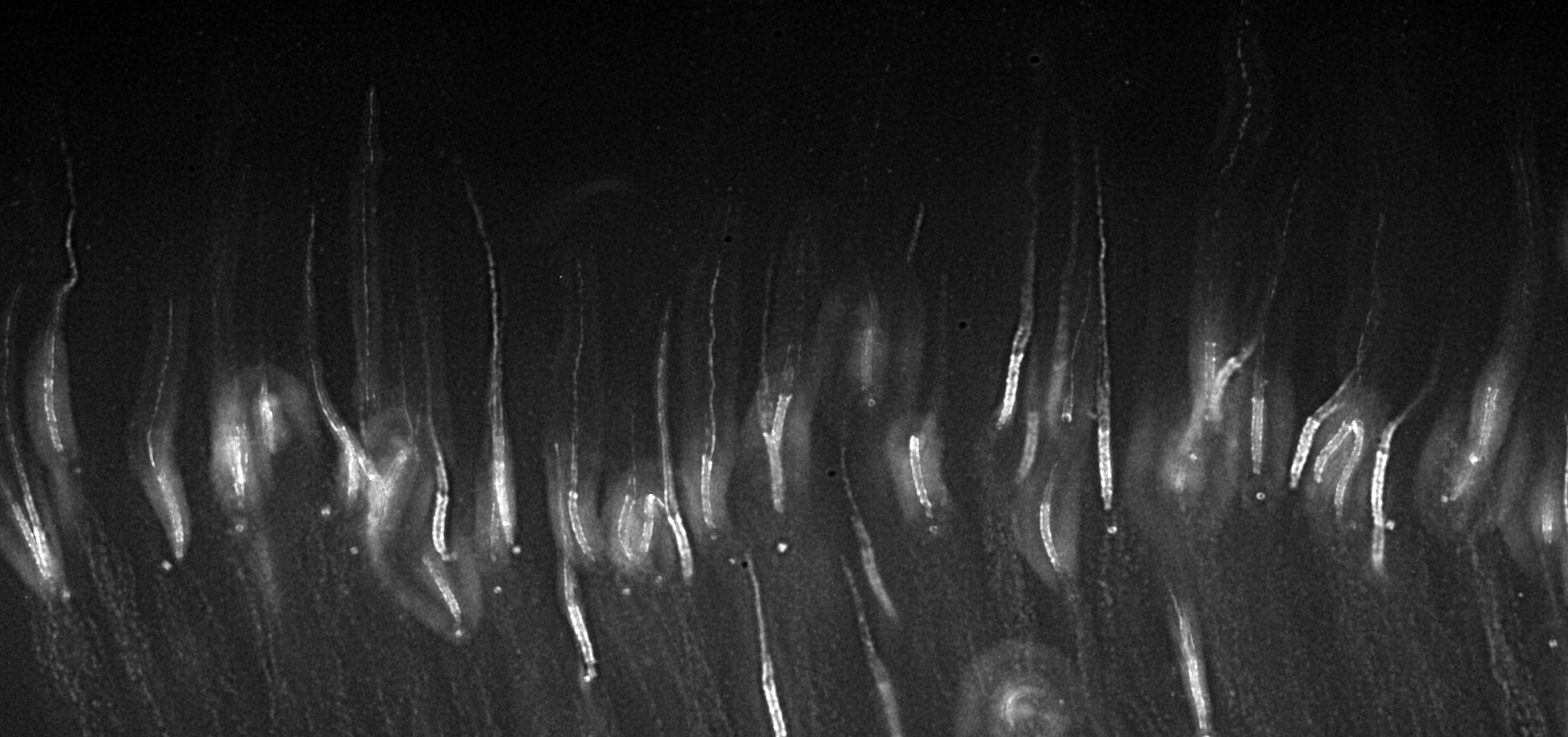

Supplement: Supplementary file 5 — Source data Fig. 3 [file 44318_2024_284_MOESM5_ESM.zip › EMBOJ-2024-118613-T _SourceDataForFigure3/3I/MAX_ATAT KO 255 GT335 TUB GP.lif - Series002_Lng_SVCC-2.tif]

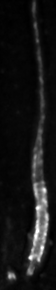

Supplement: Supplementary file 6 — Source data Fig. 4 [file 44318_2024_284_MOESM6_ESM.zip › EMBOJ-2024-118613-T _SourceDataForFigure4/4A/MAX_ATAT KO 256 18M GT335 tub .lif - Series001_Lng_SVCC-1-1.tif]

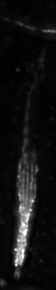

Supplement: Supplementary file 6 — Source data Fig. 4 [file 44318_2024_284_MOESM6_ESM.zip › EMBOJ-2024-118613-T _SourceDataForFigure4/4A/MAX_CCP1 KO 8M GT335 tub.lif - Series007_Lng_SVCC-1-1.tif]

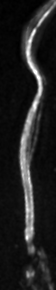

Supplement: Supplementary file 6 — Source data Fig. 4 [file 44318_2024_284_MOESM6_ESM.zip › EMBOJ-2024-118613-T _SourceDataForFigure4/4A/MAX_CCP5 KO 182 12M GT335 tub.lif - Series009_Lng_SVCC-2-1.tif]

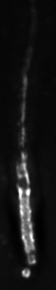

Supplement: Supplementary file 6 — Source data Fig. 4 [file 44318_2024_284_MOESM6_ESM.zip › EMBOJ-2024-118613-T _SourceDataForFigure4/4A/MAX_WT 42 15M GT335 tub.lif - Series003_Lng_SVCC-2-1-1.tif]

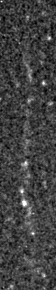

Supplement: Supplementary file 6 — Source data Fig. 4 [file 44318_2024_284_MOESM6_ESM.zip › EMBOJ-2024-118613-T _SourceDataForFigure4/4B/MAX_ATATKO 258 Ac tub.lif - Series001_Lng_SVCC-1.tif]

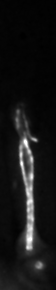

Supplement: Supplementary file 6 — Source data Fig. 4 [file 44318_2024_284_MOESM6_ESM.zip › EMBOJ-2024-118613-T _SourceDataForFigure4/4B/MAX_CCP1 KO 1180 ac tubtub.lif - Series002_Lng_SVCC-1-1.tif]

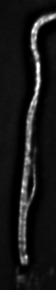

Supplement: Supplementary file 6 — Source data Fig. 4 [file 44318_2024_284_MOESM6_ESM.zip › EMBOJ-2024-118613-T _SourceDataForFigure4/4B/MAX_CCP5 KO 78 12M Ac tub tub .lif - Series002_Lng_SVCC-2-1.tif]

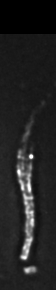

Supplement: Supplementary file 6 — Source data Fig. 4 [file 44318_2024_284_MOESM6_ESM.zip › EMBOJ-2024-118613-T _SourceDataForFigure4/4B/MAX_WT adult acetylated tub and tub.lif - Series001_Lng_SVCC-2.tif]

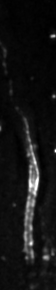

Supplement: Supplementary file 6 — Source data Fig. 4 [file 44318_2024_284_MOESM6_ESM.zip › EMBOJ-2024-118613-T _SourceDataForFigure4/4C/MAX_ATAT KO 255 TAP952 TUB GP.lif - Series002_Lng_SVCC-1-1.tif]

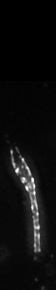

Supplement: Supplementary file 6 — Source data Fig. 4 [file 44318_2024_284_MOESM6_ESM.zip › EMBOJ-2024-118613-T _SourceDataForFigure4/4C/MAX_CCP1 KO 8M TAP952 tub.lif - Series004_Lng_SVCC-1.tif]

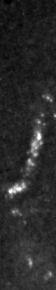

Supplement: Supplementary file 6 — Source data Fig. 4 [file 44318_2024_284_MOESM6_ESM.zip › EMBOJ-2024-118613-T _SourceDataForFigure4/4C/MAX_CCP5 KO 78 12M TAP952 tub .lif - Series003_Lng_SVCC-1.tif]

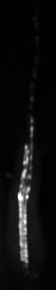

Supplement: Supplementary file 6 — Source data Fig. 4 [file 44318_2024_284_MOESM6_ESM.zip › EMBOJ-2024-118613-T _SourceDataForFigure4/4C/MAX_WT 42 15M tap952 tub.lif - Series004_Lng_SVCC-1-1-1.tif]

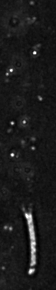

Supplement: Supplementary file 6 — Source data Fig. 4 [file 44318_2024_284_MOESM6_ESM.zip › EMBOJ-2024-118613-T _SourceDataForFigure4/4D/MAX_20210923 CCP1 KO 8M poc5 tub.lif - Series004_Lng_SVCC-1-1.tif]

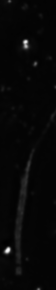

Supplement: Supplementary file 6 — Source data Fig. 4 [file 44318_2024_284_MOESM6_ESM.zip › EMBOJ-2024-118613-T _SourceDataForFigure4/4D/MAX_20220929 CCP5 KO 12M poc5tub .lif - Series006_Lng_SVCC-1-1.tif]

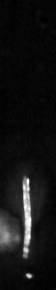

Supplement: Supplementary file 6 — Source data Fig. 4 [file 44318_2024_284_MOESM6_ESM.zip › EMBOJ-2024-118613-T _SourceDataForFigure4/4D/MAX_ATAT KO 256 18M poc5 tub .lif - Series001_Lng_SVCC-1.tif]

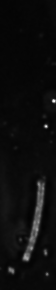

Supplement: Supplementary file 6 — Source data Fig. 4 [file 44318_2024_284_MOESM6_ESM.zip › EMBOJ-2024-118613-T _SourceDataForFigure4/4D/MAX_T3T8 HET 704 - poc5.lif - Series003_Lng_SVCC-1-1.tif]

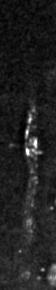

Supplement: Supplementary file 6 — Source data Fig. 4 [file 44318_2024_284_MOESM6_ESM.zip › EMBOJ-2024-118613-T _SourceDataForFigure4/4E/MAX_ATAT KO 255 LCA5 TUB GP.lif - Series001_Lng_SVCC-1-1.tif]

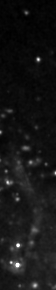

Supplement: Supplementary file 6 — Source data Fig. 4 [file 44318_2024_284_MOESM6_ESM.zip › EMBOJ-2024-118613-T _SourceDataForFigure4/4E/MAX_CCP1 KO 1180 LCA5 tub.lif - Series003_Lng_SVCC-1.tif]

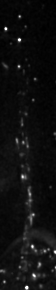

Supplement: Supplementary file 6 — Source data Fig. 4 [file 44318_2024_284_MOESM6_ESM.zip › EMBOJ-2024-118613-T _SourceDataForFigure4/4E/MAX_CCP5 KO 78 12M LCA5 tub .lif - Series007_Lng_SVCC-1-1.tif]

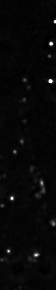

Supplement: Supplementary file 6 — Source data Fig. 4 [file 44318_2024_284_MOESM6_ESM.zip › EMBOJ-2024-118613-T _SourceDataForFigure4/4E/MAX_TTLL3 8 het 704 Lca5 tub.lif - Series005_Lng_SVCC-2-1.tif]

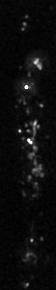

Supplement: Supplementary file 6 — Source data Fig. 4 [file 44318_2024_284_MOESM6_ESM.zip › EMBOJ-2024-118613-T _SourceDataForFigure4/4F/MAX_20210923 CCP1 KO 8M IFT88 tub.lif - Series004_Lng_SVCC-1.tif]

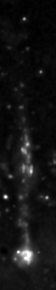

Supplement: Supplementary file 6 — Source data Fig. 4 [file 44318_2024_284_MOESM6_ESM.zip › EMBOJ-2024-118613-T _SourceDataForFigure4/4F/MAX_ATAT KO 255 IFT88 tub.lif - Series002_Lng_SVCC-1.tif]

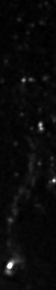

Supplement: Supplementary file 6 — Source data Fig. 4 [file 44318_2024_284_MOESM6_ESM.zip › EMBOJ-2024-118613-T _SourceDataForFigure4/4F/MAX_CCP5 KO 12M IFT88 tub.lif - Series004_Lng_SVCC-2-1.tif]

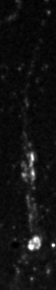

Supplement: Supplementary file 6 — Source data Fig. 4 [file 44318_2024_284_MOESM6_ESM.zip › EMBOJ-2024-118613-T _SourceDataForFigure4/4F/MAX_CCP5 WT 18M IFT 88tub.lif - Series003_Lng_SVCC-1.tif]

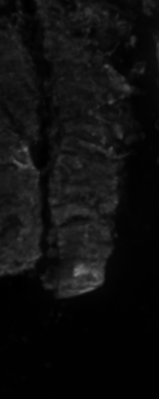

Supplement: Supplementary file 7 — Source data Fig. 5 [file 44318_2024_284_MOESM7_ESM.zip › EMBOJ-2024-118613-T _SourceDataForFigure5/5A/MAX_CCP5 KO 3M rhodo tub.lif - Series001_Lng_SVCC-2-1.tif]

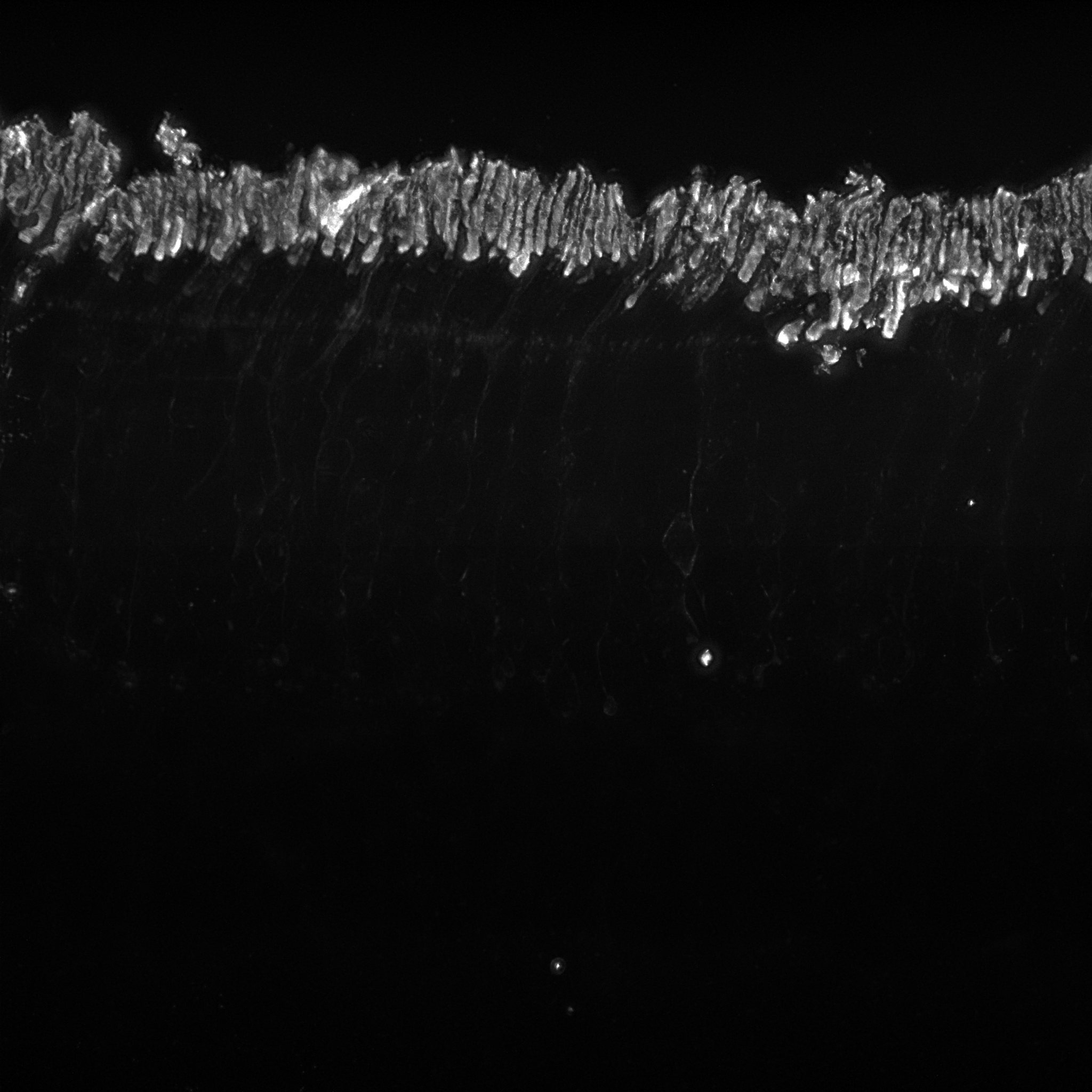

Supplement: Supplementary file 7 — Source data Fig. 5 [file 44318_2024_284_MOESM7_ESM.zip › EMBOJ-2024-118613-T _SourceDataForFigure5/5A/MAX_CCP5 KO rhodo tub.lif - Series002_Lng_SVCC.tif]

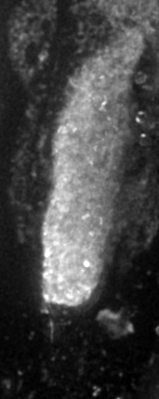

Supplement: Supplementary file 7 — Source data Fig. 5 [file 44318_2024_284_MOESM7_ESM.zip › EMBOJ-2024-118613-T _SourceDataForFigure5/5B/MAX_c5ko 292 7M rhodo tub.lif - Series001_Lng_SVCC-1-1-1.tif]

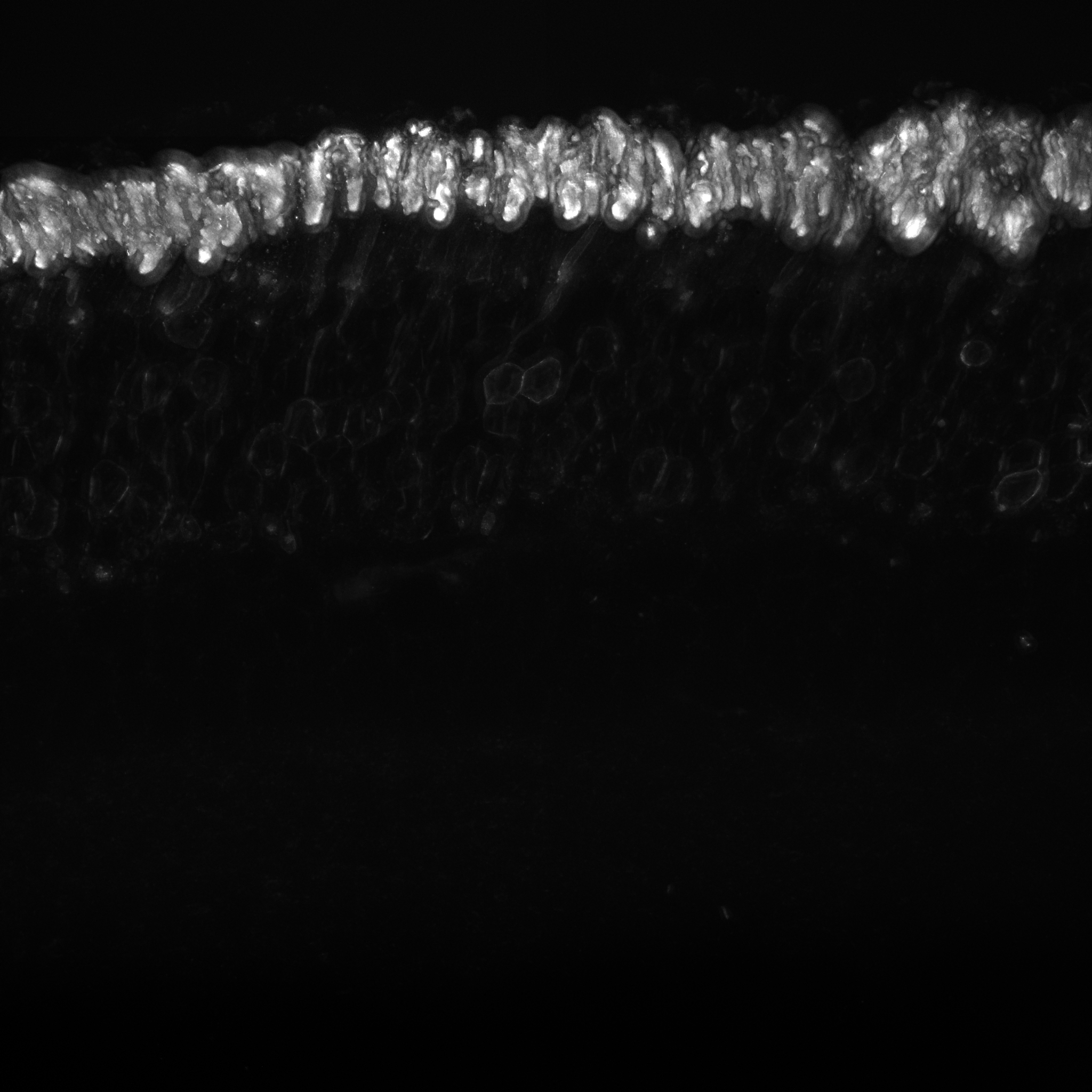

Supplement: Supplementary file 7 — Source data Fig. 5 [file 44318_2024_284_MOESM7_ESM.zip › EMBOJ-2024-118613-T _SourceDataForFigure5/5B/MAX_CCP5 KO 299 rhodo tub.lif - Series004_Lng_SVCC.tif]

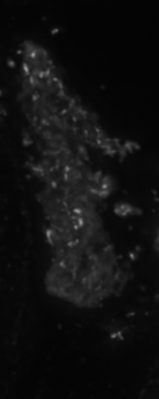

Supplement: Supplementary file 7 — Source data Fig. 5 [file 44318_2024_284_MOESM7_ESM.zip › EMBOJ-2024-118613-T _SourceDataForFigure5/5C/MAX_c5ko 255 10M rhodo tub.lif - Series006_Lng_SVCC-1-1.tif]

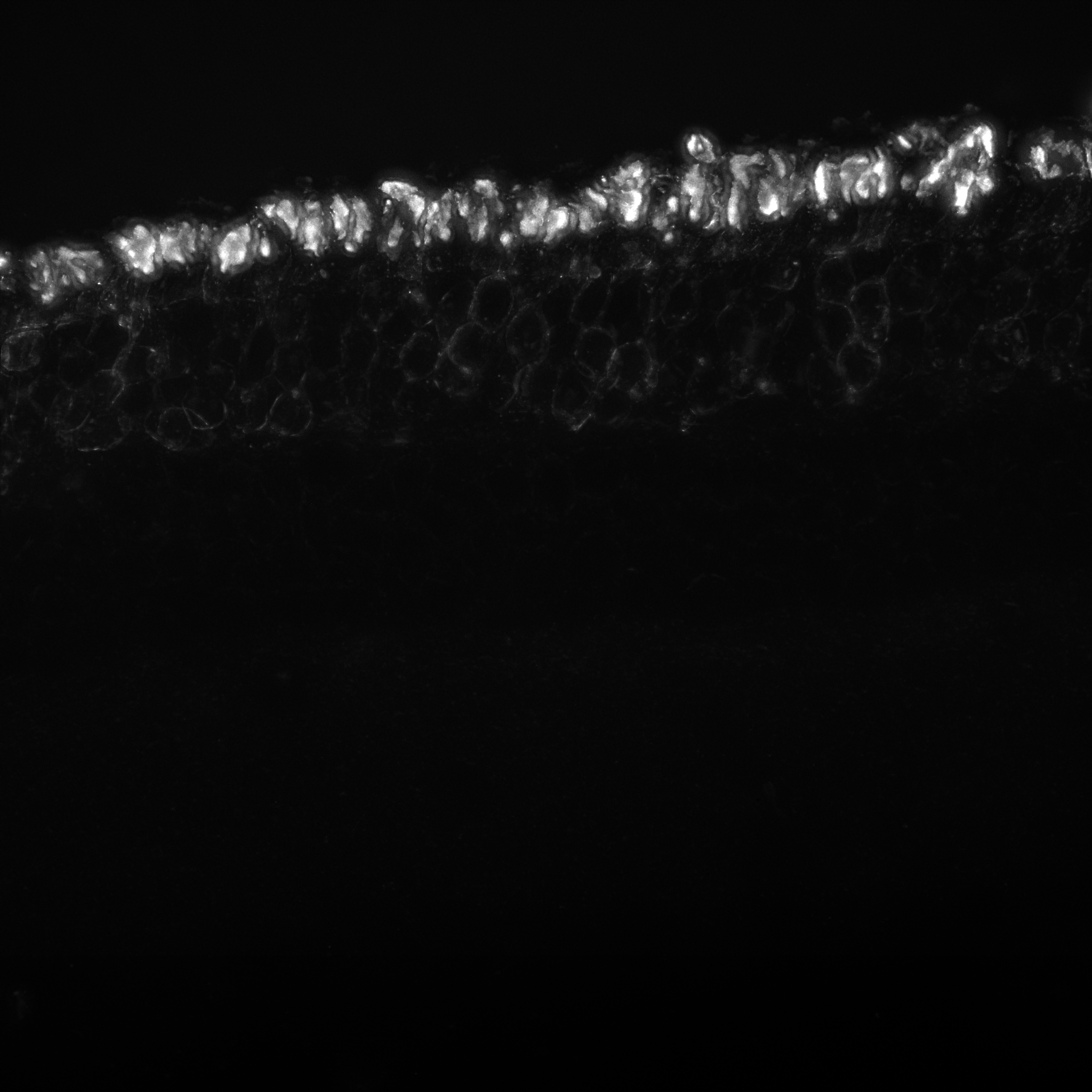

Supplement: Supplementary file 7 — Source data Fig. 5 [file 44318_2024_284_MOESM7_ESM.zip › EMBOJ-2024-118613-T _SourceDataForFigure5/5C/MAX_CCP5 KO 255 rhodo tub.lif - Series002_Lng_SVCC.tif]

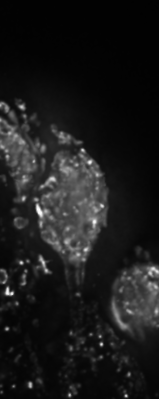

Supplement: Supplementary file 7 — Source data Fig. 5 [file 44318_2024_284_MOESM7_ESM.zip › EMBOJ-2024-118613-T _SourceDataForFigure5/5D/MAX_c5ko 182 12M rhodo tub.lif - Series006_Lng_SVCC-1-1.tif]

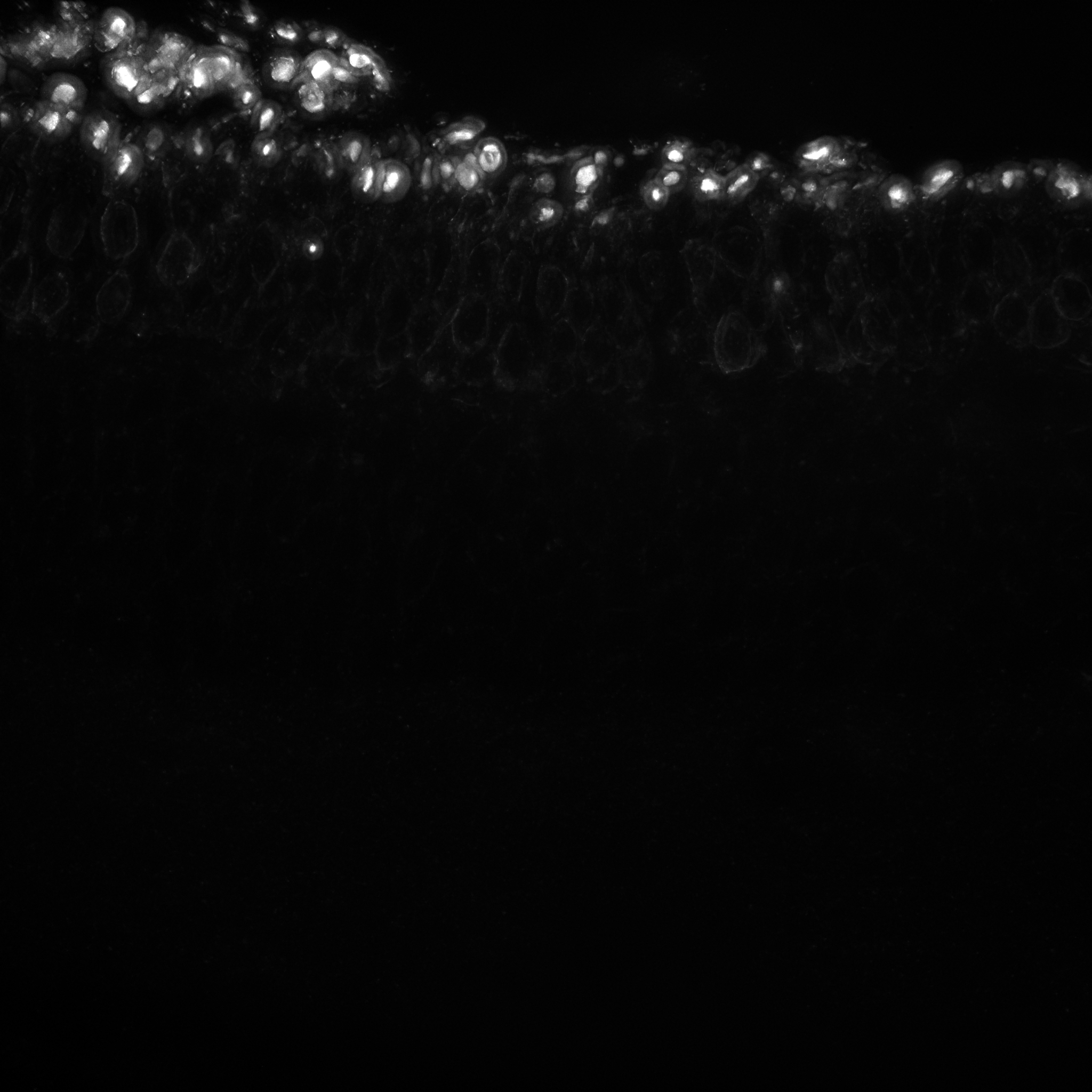

Supplement: Supplementary file 7 — Source data Fig. 5 [file 44318_2024_284_MOESM7_ESM.zip › EMBOJ-2024-118613-T _SourceDataForFigure5/5D/MAX_CCP5 KO 182 rhodo tub.lif - Series002_Lng_SVCC.tif]

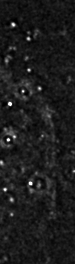

Supplement: Supplementary file 7 — Source data Fig. 5 [file 44318_2024_284_MOESM7_ESM.zip › EMBOJ-2024-118613-T _SourceDataForFigure5/5F/MAX_CCP5 KO 18M tap952 tub.lif - Series002_000_Lng_SVCC-1-1.tif]

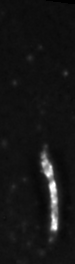

Supplement: Supplementary file 7 — Source data Fig. 5 [file 44318_2024_284_MOESM7_ESM.zip › EMBOJ-2024-118613-T _SourceDataForFigure5/5F/MAX_CCP5 KO TAP952 tub.lif - Series002_Lng_SVCC-1-1.tif]

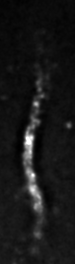

Supplement: Supplementary file 7 — Source data Fig. 5 [file 44318_2024_284_MOESM7_ESM.zip › EMBOJ-2024-118613-T _SourceDataForFigure5/5F/MAX_CCP5KO 259 tap952 tub.lif - Series001_Lng_SVCC-1-1-1.tif]

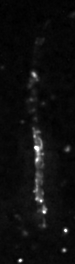

Supplement: Supplementary file 7 — Source data Fig. 5 [file 44318_2024_284_MOESM7_ESM.zip › EMBOJ-2024-118613-T _SourceDataForFigure5/5F/MAX_CCP5KO 292 tap952 tub.lif - Series006_Lng_SVCC-1-4-1.tif]

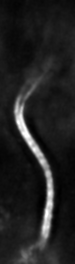

Supplement: Supplementary file 7 — Source data Fig. 5 [file 44318_2024_284_MOESM7_ESM.zip › EMBOJ-2024-118613-T _SourceDataForFigure5/5G/MAX_CCP5 KO 182 12M GT335 tub.lif - Series001_Lng_SVCC-1.tif]

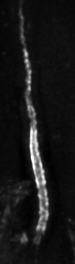

Supplement: Supplementary file 7 — Source data Fig. 5 [file 44318_2024_284_MOESM7_ESM.zip › EMBOJ-2024-118613-T _SourceDataForFigure5/5G/MAX_CCP5 KO GT335 tub.lif - Series003_Lng_SVCC-2-1.tif]

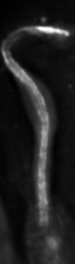

Supplement: Supplementary file 7 — Source data Fig. 5 [file 44318_2024_284_MOESM7_ESM.zip › EMBOJ-2024-118613-T _SourceDataForFigure5/5G/MAX_CCP5KO 255 GT335 tub.lif - Series002_Lng_SVCC-1-1.tif]

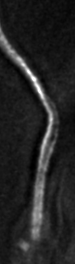

Supplement: Supplementary file 7 — Source data Fig. 5 [file 44318_2024_284_MOESM7_ESM.zip › EMBOJ-2024-118613-T _SourceDataForFigure5/5G/MAX_CCP5KO 292 GT335 tub.lif - Series003_Lng_SVCC-1-3-1.tif]

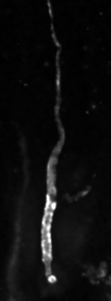

Supplement: Supplementary file 7 — Source data Fig. 5 [file 44318_2024_284_MOESM7_ESM.zip › EMBOJ-2024-118613-T _SourceDataForFigure5/5J/MAX_WT 41 15M GT335 tub.lif - Series003_Lng_SVCC-1-1-1.tif]

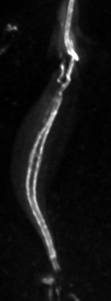

Supplement: Supplementary file 7 — Source data Fig. 5 [file 44318_2024_284_MOESM7_ESM.zip › EMBOJ-2024-118613-T _SourceDataForFigure5/5K/MAX_CCP5 KO 182 12M GT335 tub.lif - Series007_Lng_SVCC-1-1.tif]

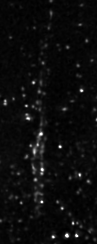

Supplement: Supplementary file 7 — Source data Fig. 5 [file 44318_2024_284_MOESM7_ESM.zip › EMBOJ-2024-118613-T _SourceDataForFigure5/5O/MAX_WT 118 RPGR tub.lif - Series013_Lng_SVCC-1-1.tif]

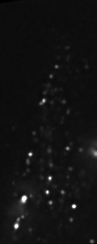

Supplement: Supplementary file 7 — Source data Fig. 5 [file 44318_2024_284_MOESM7_ESM.zip › EMBOJ-2024-118613-T _SourceDataForFigure5/5P/MAX_CCP5 KO 12M 04.2023 RPGR tub.lif - Series006_Lng_SVCC-2-1.tif]

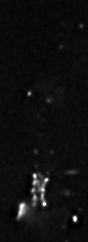

Supplement: Supplementary file 7 — Source data Fig. 5 [file 44318_2024_284_MOESM7_ESM.zip › EMBOJ-2024-118613-T _SourceDataForFigure5/5R/AVG_Human retina RPGR sigma tub.lif - Series007_Lng_SVCC-1.tif]

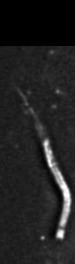

Supplement: Supplementary file 8 — Source data Fig. 6 [file 44318_2024_284_MOESM8_ESM.zip › EMBOJ-2024-118613-T _SourceDataForFigure6/6A/CCP5 ko 12M n2 poc5.lif - Series004_Lng_SVCC-1-2-1-1.tif]

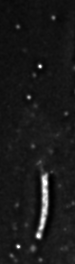

Supplement: Supplementary file 8 — Source data Fig. 6 [file 44318_2024_284_MOESM8_ESM.zip › EMBOJ-2024-118613-T _SourceDataForFigure6/6A/MAX_CCP5 KO poc5 tub.lif - Series002_Lng_SVCC-1-2-1-1.tif]

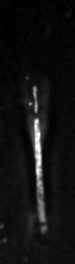

Supplement: Supplementary file 8 — Source data Fig. 6 [file 44318_2024_284_MOESM8_ESM.zip › EMBOJ-2024-118613-T _SourceDataForFigure6/6A/MAX_CCP5KO 255 POC5 tub.lif - Series005_Lng_SVCC-1-1.tif]

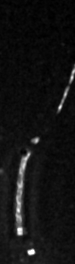

Supplement: Supplementary file 8 — Source data Fig. 6 [file 44318_2024_284_MOESM8_ESM.zip › EMBOJ-2024-118613-T _SourceDataForFigure6/6A/MAX_CCP5KO 292 poc5 tub.lif - Series004_Lng_SVCC-1-1.tif]

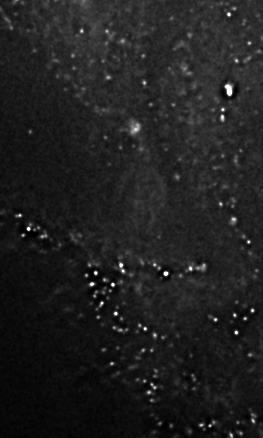

Supplement: Supplementary file 8 — Source data Fig. 6 [file 44318_2024_284_MOESM8_ESM.zip › EMBOJ-2024-118613-T _SourceDataForFigure6/6B/CCP5 ko 12M n2 IFT88.lif - Series001_Lng_SVCC-1-2.tif]

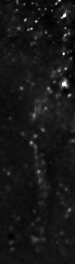

Supplement: Supplementary file 8 — Source data Fig. 6 [file 44318_2024_284_MOESM8_ESM.zip › EMBOJ-2024-118613-T _SourceDataForFigure6/6B/MAX_CCP5 KO 255 IFT88 tub.lif - Series002_Lng_SVCC-1-1.tif]

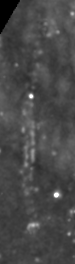

Supplement: Supplementary file 8 — Source data Fig. 6 [file 44318_2024_284_MOESM8_ESM.zip › EMBOJ-2024-118613-T _SourceDataForFigure6/6B/MAX_CCP5 KO 292 IFT88 tub.lif - Series001_Lng_SVCC-1-2-1.tif]

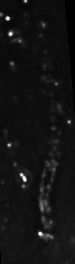

Supplement: Supplementary file 8 — Source data Fig. 6 [file 44318_2024_284_MOESM8_ESM.zip › EMBOJ-2024-118613-T _SourceDataForFigure6/6B/MAX_CCP5 KO IFT88 tub.lif - Series003_Lng_SVCC-1-1.tif]

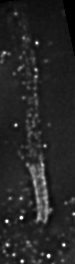

Supplement: Supplementary file 8 — Source data Fig. 6 [file 44318_2024_284_MOESM8_ESM.zip › EMBOJ-2024-118613-T _SourceDataForFigure6/6E/AVG_CCP5 WT CEP290 tub.lif - Series002_Lng_SVCC-1.tif]

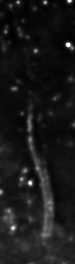

Supplement: Supplementary file 8 — Source data Fig. 6 [file 44318_2024_284_MOESM8_ESM.zip › EMBOJ-2024-118613-T _SourceDataForFigure6/6E/AVG_ko 262 cep290 tub.lif - Series003_Lng_SVCC-1-5-2.tif]

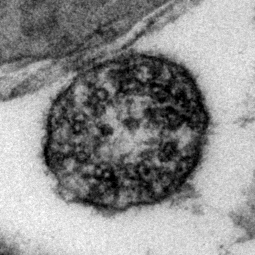

Supplement: Supplementary file 8 — Source data Fig. 6 [file 44318_2024_284_MOESM8_ESM.zip › EMBOJ-2024-118613-T _SourceDataForFigure6/6G/019_42kx-1.tif]

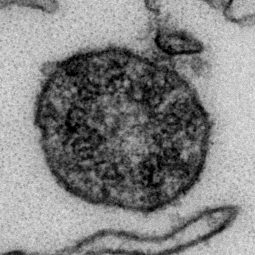

Supplement: Supplementary file 8 — Source data Fig. 6 [file 44318_2024_284_MOESM8_ESM.zip › EMBOJ-2024-118613-T _SourceDataForFigure6/6G/061_42kx-1.tif]
