# Supplementary figures and images for: Glutamylation imbalance impairs the molecular architecture of the photoreceptor cilium (part 2 of 2)
Source: EMBO J. 2024 Nov 11;43(24):19. doi: 10.1038/s44318-024-00284-1 (PMC11649768; doi:10.1038/s44318-024-00284-1)

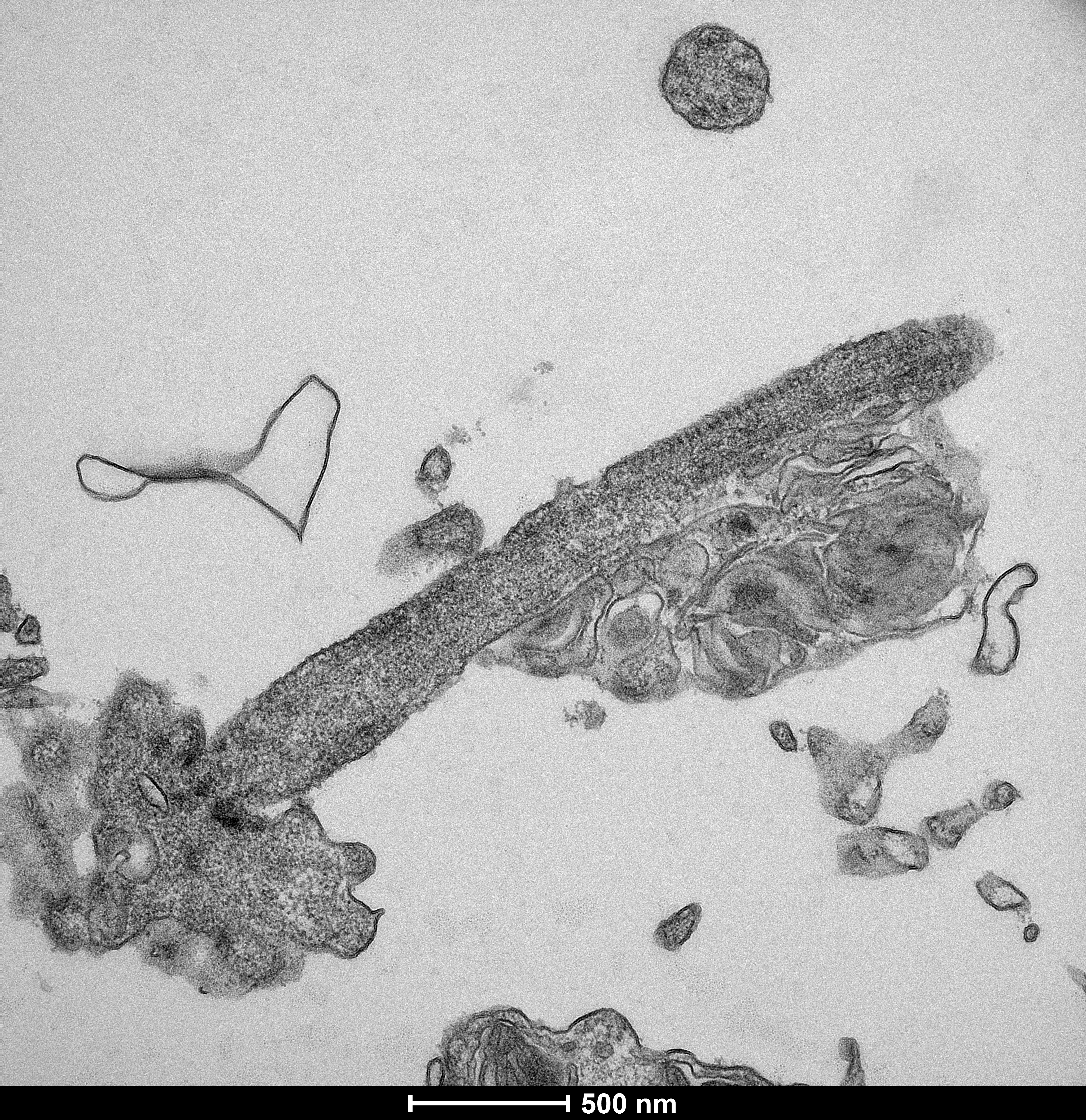

Supplement: Supplementary file 8 — Source data Fig. 6 [file 44318_2024_284_MOESM8_ESM.zip › EMBOJ-2024-118613-T _SourceDataForFigure6/6H/28.tif]

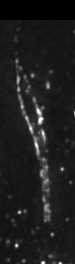

Supplement: Supplementary file 8 — Source data Fig. 6 [file 44318_2024_284_MOESM8_ESM.zip › EMBOJ-2024-118613-T _SourceDataForFigure6/6I/MAX_CCP5 KO 12M 042023 MAP9 tub.lif - Series018_Lng_SVCC-1-1-1.tif]

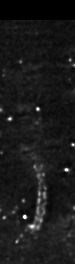

Supplement: Supplementary file 8 — Source data Fig. 6 [file 44318_2024_284_MOESM8_ESM.zip › EMBOJ-2024-118613-T _SourceDataForFigure6/6I/MAX_CCP5 WT 20M 20221107MAP9.lif - Series006_Lng_SVCC-1-2-1-1.tif]

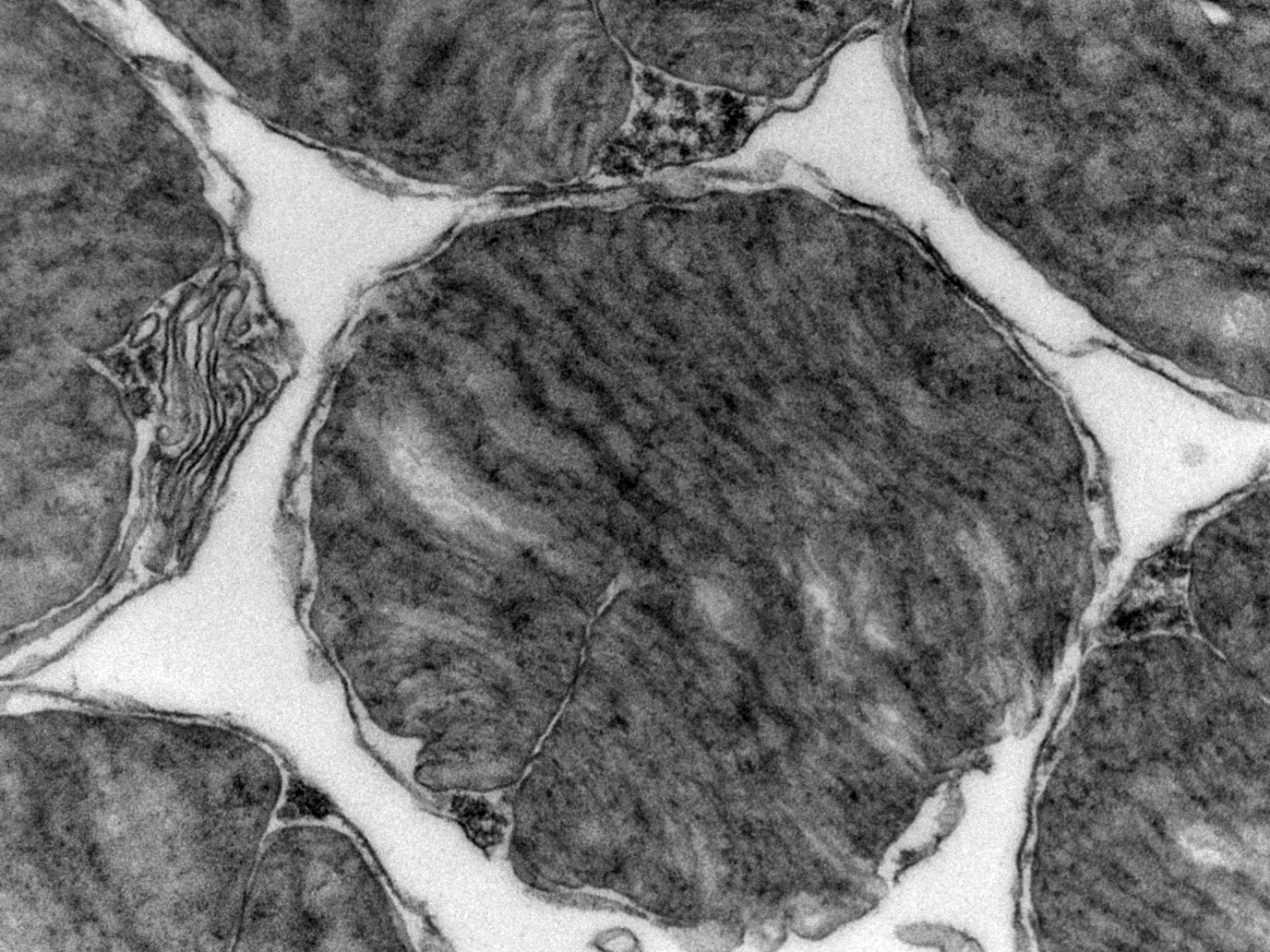

Supplement: Supplementary file 8 — Source data Fig. 6 [file 44318_2024_284_MOESM8_ESM.zip › EMBOJ-2024-118613-T _SourceDataForFigure6/6K/016_42kx.TIF]

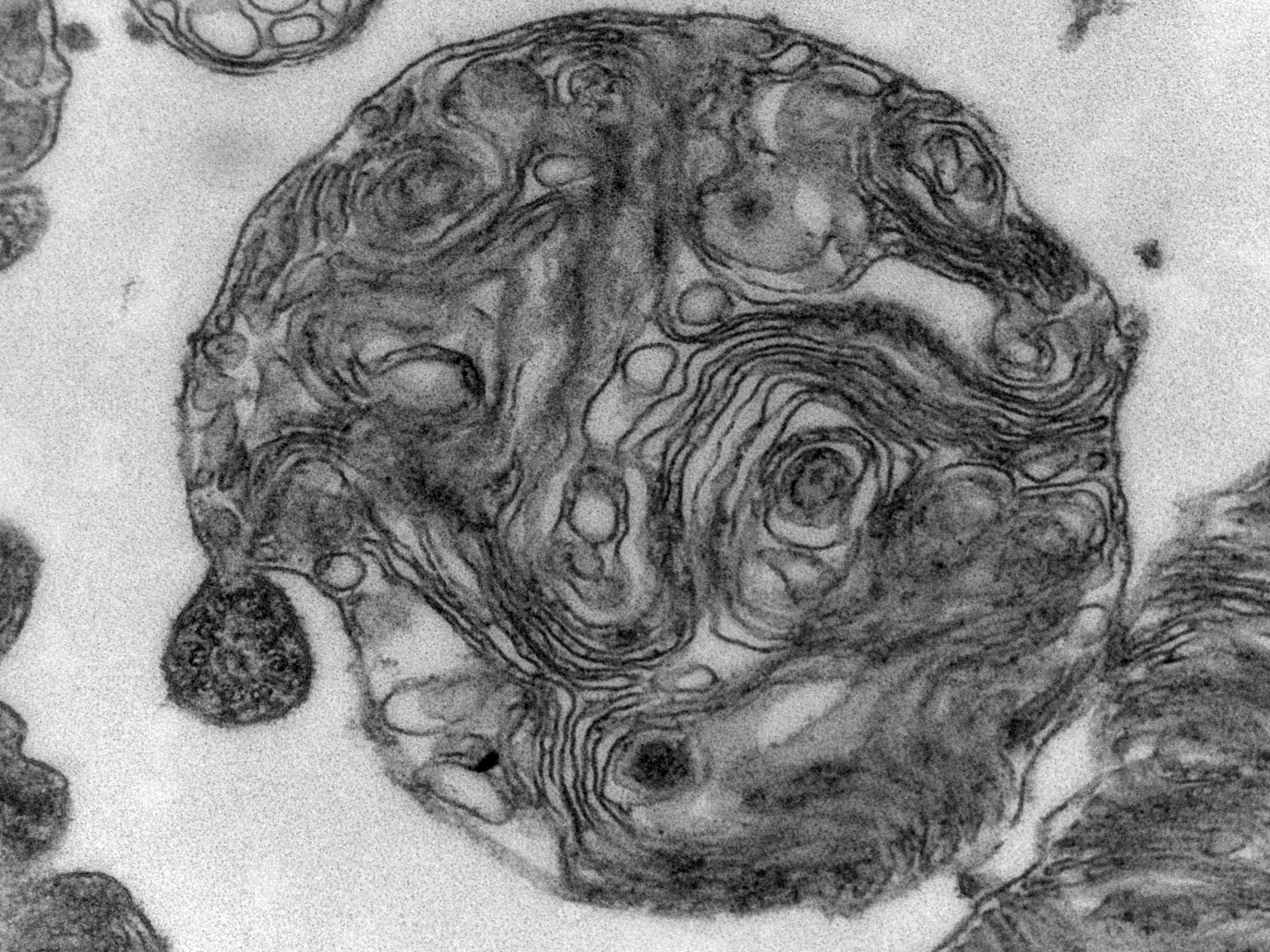

Supplement: Supplementary file 8 — Source data Fig. 6 [file 44318_2024_284_MOESM8_ESM.zip › EMBOJ-2024-118613-T _SourceDataForFigure6/6L/036_42kx.TIF]
