# Supplementary material for: Glutamylation imbalance impairs the molecular architecture of the photoreceptor cilium
Source: EMBO J. 2024 Nov 11;43(24):19. doi: 10.1038/s44318-024-00284-1 (PMC11649768; doi:10.1038/s44318-024-00284-1)
Supplement: Supplementary file 9 — Expanded View Figures [file 44318_2024_284_MOESM9_ESM.pdf]

Expanded View Figures

Figure EV1. Architecture of the photoreceptor outer segment.

(**A**) Expanded photoreceptor cell layer of a WT mouse retina stained for tubulin (magenta) and rhodopsin (green). On the right, inset of a single photoreceptor cell outer segment stained for tubulin, revealing the different regions of the cilium. Scale bars: left: 10 µm; right: 500 nm. (**B**) Quantification of the expansion factor (EF) used for the whole study. EF = 4.248 +/− 0.588 (mean +/− SD) (*n* = 55; *N* > 5 animals). (**C**) Model explaining the tubulin code and highlighting the PTMs analyzed in this study. (**D**) 18-month-old WT expanded photoreceptor cell stained for glutamylation (GT335, cyan) and tubulin (magenta) highlighting differences in GT335 staining along the OS. Scale bar: 500 nm. (**E**) Zoom in on the 3 different OS subregions of GT335 staining analyzed (Centriole, CC, Bulge). Scale bar: 200 nm. (**F**) Quantification of GT335 signal intensity in the centriole, the CC and the bulge region, normalized on background. Centriole: 5.55 +/− 3.61 (*n* = 38); CC: 15.12 +/− 12.67 (*n* = 39); Bulge: 8.08 +/− 6.00 (*n* = 39) (*N* = 3 animals) (mean +/− SD). Test: Kruskal–Wallis with Dunn's multiple comparison. centriole vs. CC: ****(adjusted *P* value: <0.0001); centriole vs. bulge: ns (adjusted *P* value: 0.3331); CC vs. bulge: *(adjusted *P* value: 0.0272). Each animal corresponds to one experimental replicate.

© The Author(s)

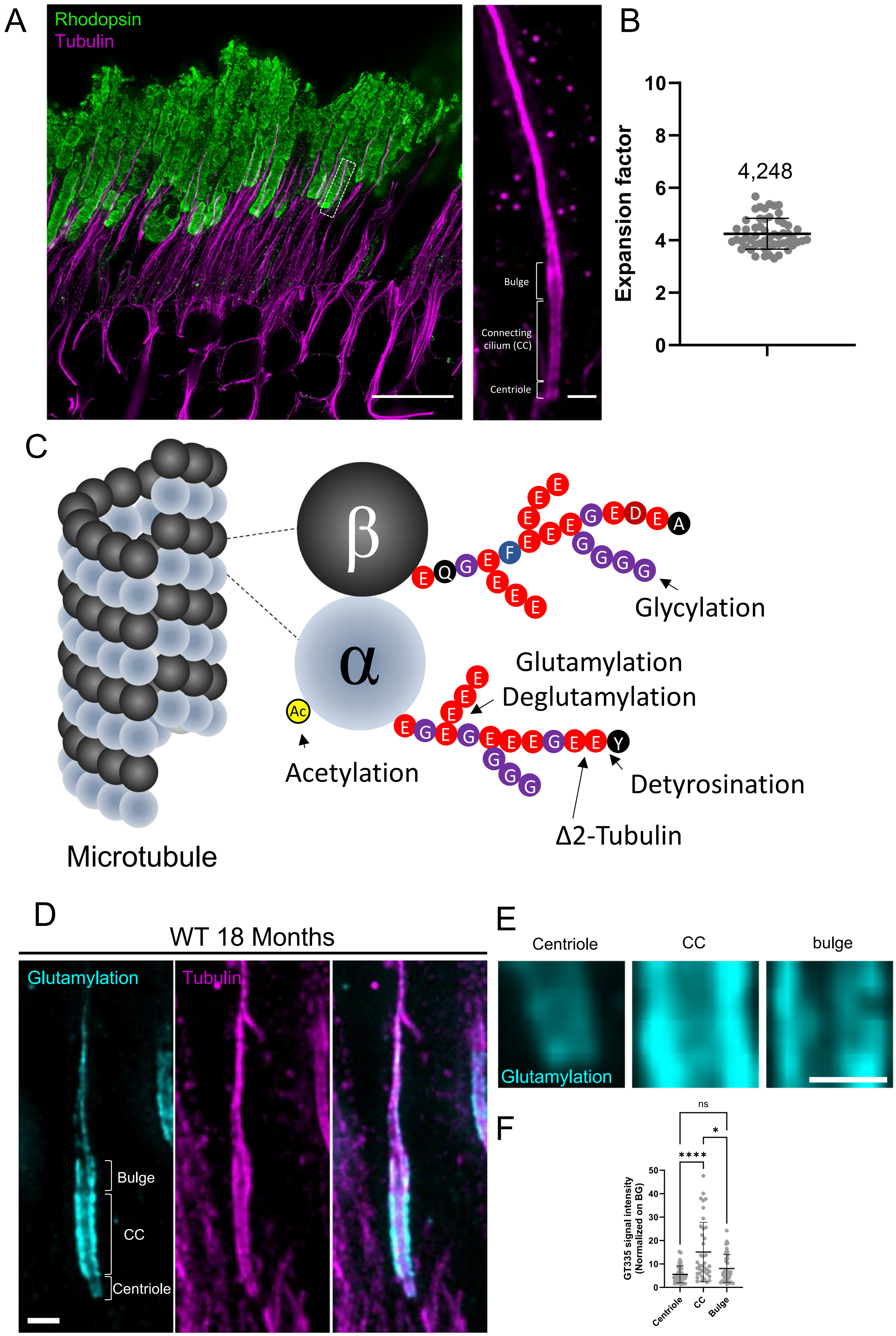
A
Rhodopsin
Tubulin
Bulge
Connecting cilium (CC)
Centriole
B
Expansion factor
4,248
10
8
6
4
2
0
C
β
α
Glycylation
Glutamylation
Deglutamylation
Acetylation
Detyrosination
Δ2-Tubulin
Microtubule
D
WT 18 Months
Glutamylation
Tubulin
Bulge
CC
Centriole
E
Centriole
CC
bulge
Glutamylation
F
GT335 signal intensity (Normalized on BG)
ns
*
****
Centriole
CC
Bulge

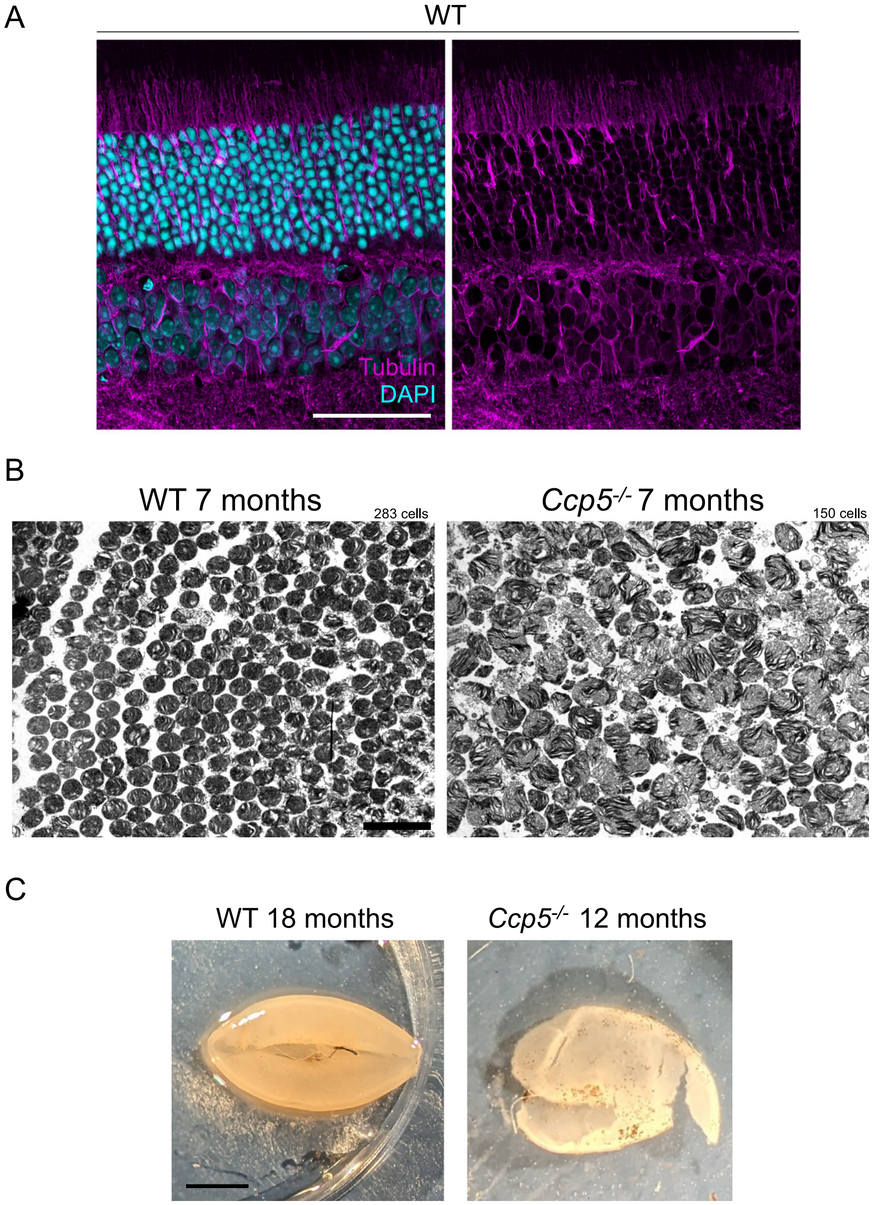

Figure EV2. Global morphology of WT and *Ccp5*$^{-/-}$ retinas.

(**A**) Expanded WT mouse retina stained for tubulin (magenta) and DAPI (cyan). Note that ONL thickness can be measured only with tubulin staining, where nuclei position is clearly visible. Scale bar: 50 µm. (**B**) EM micrographs of 7-month-old WT or *Ccp5*$^{-/-}$ retinas at low magnification to highlight defects in the organization of the membrane discs together with an important decrease in the number of cells (quantified on the top right). Scale bar: 5 µm. (**C**) 12-month-old WT (left) and *Ccp5*$^{-/-}$ (right) retinas during dissection. Note that *Ccp5*$^{-/-}$ retina is thinner and pigmented, reflecting a strong degeneration, a feature that we already observed previously (Faber et al, 2023). Scale bar: 1 mm.

 © The Author(s)

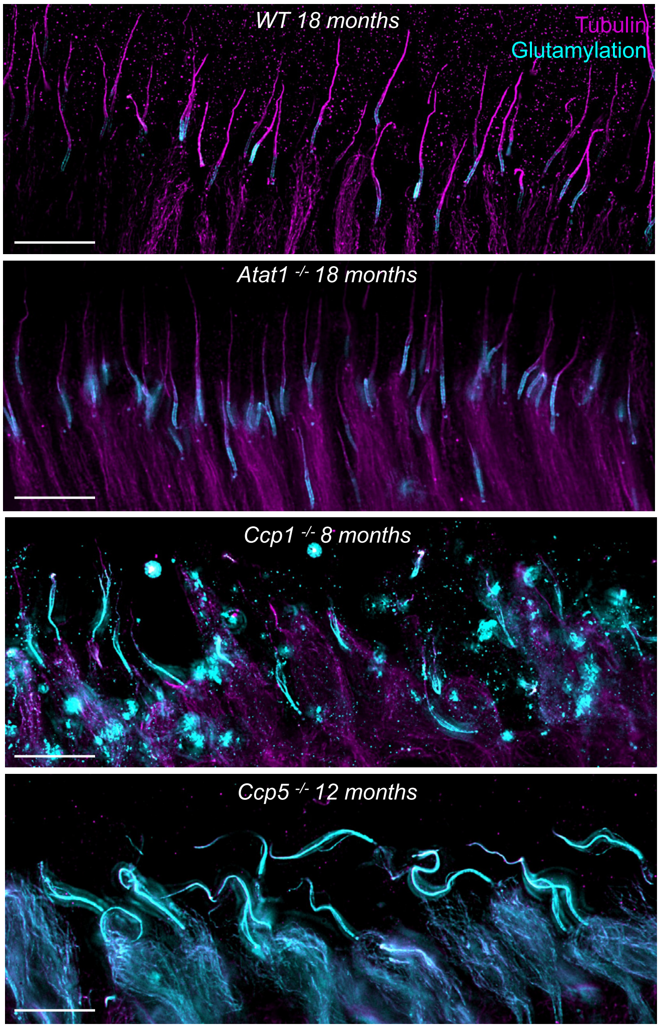

Figure EV3. Glutamylation level observed in several PTM mutants.

Large field of view of WT, *Atat1*$^{-/-}$, *Ccp1*$^{-/-}$ and *Ccp5*$^{-/-}$ expanded photoreceptor cell stained with GT335 (cyan) and tubulin (magenta). Note the intense glutamylation signal inside photoreceptor cell bodies in *Ccp5*$^{-/-}$ retina. Scale bar: 5 µm.

© The Author(s)

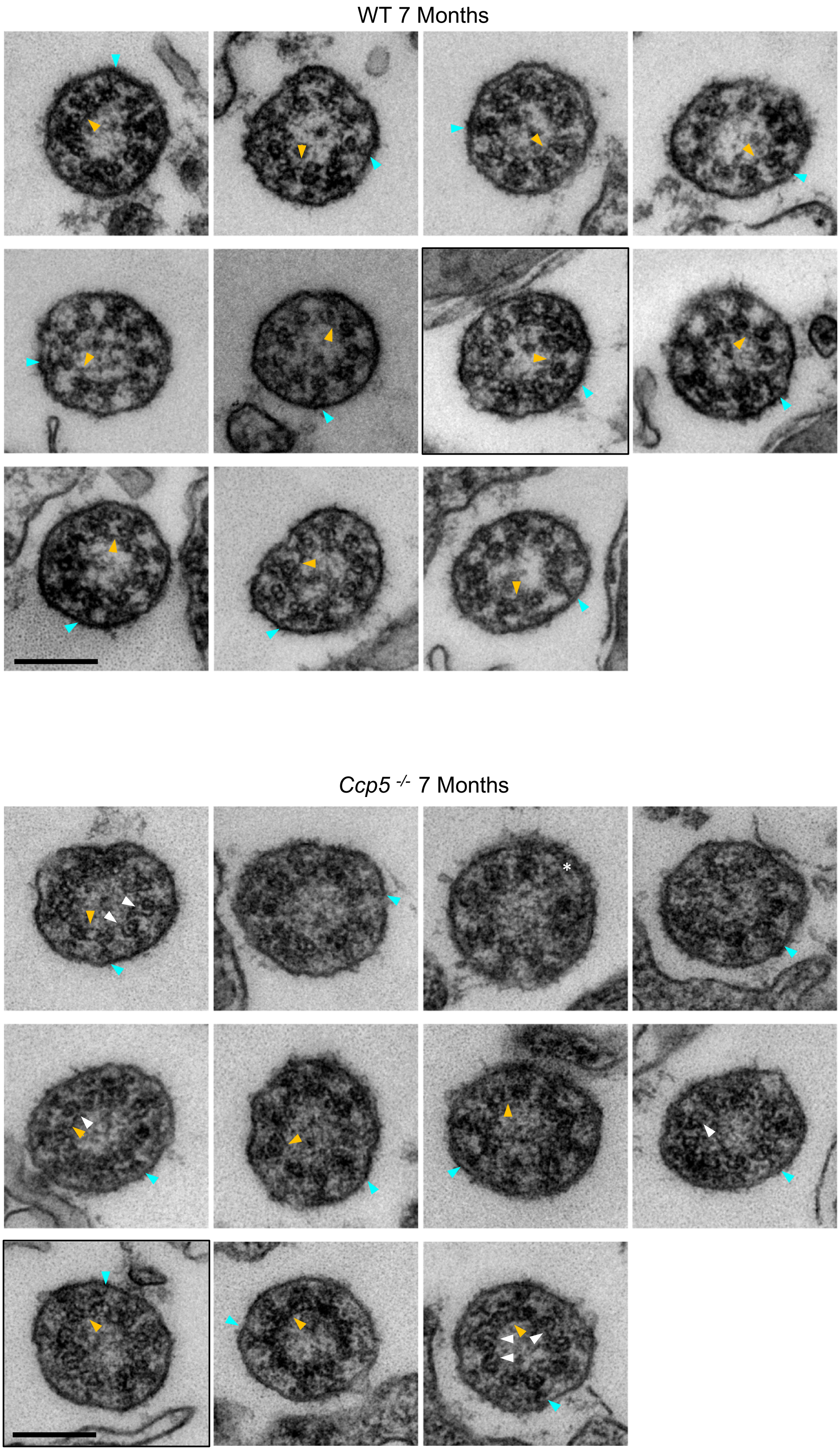
WT 7 Months
Ccp5 -/- 7 Months

Figure EV4. EM gallery of 7-month-old WT and *Ccp5*$^{-/-}$ photoreceptor CC.

EM micrographs of 7-month-old WT and Ccp5−/− photoreceptor CC observed in transverse sections. White arrowheads highlight open B-tubules. Asterix depicts missing MTD. Cyan and orange arrowheads show Y-links and inner scaffold, respectively. The two images with black border are the ones used in the Fig. 6. Scale bar: 200 nm.

© The Author(s)

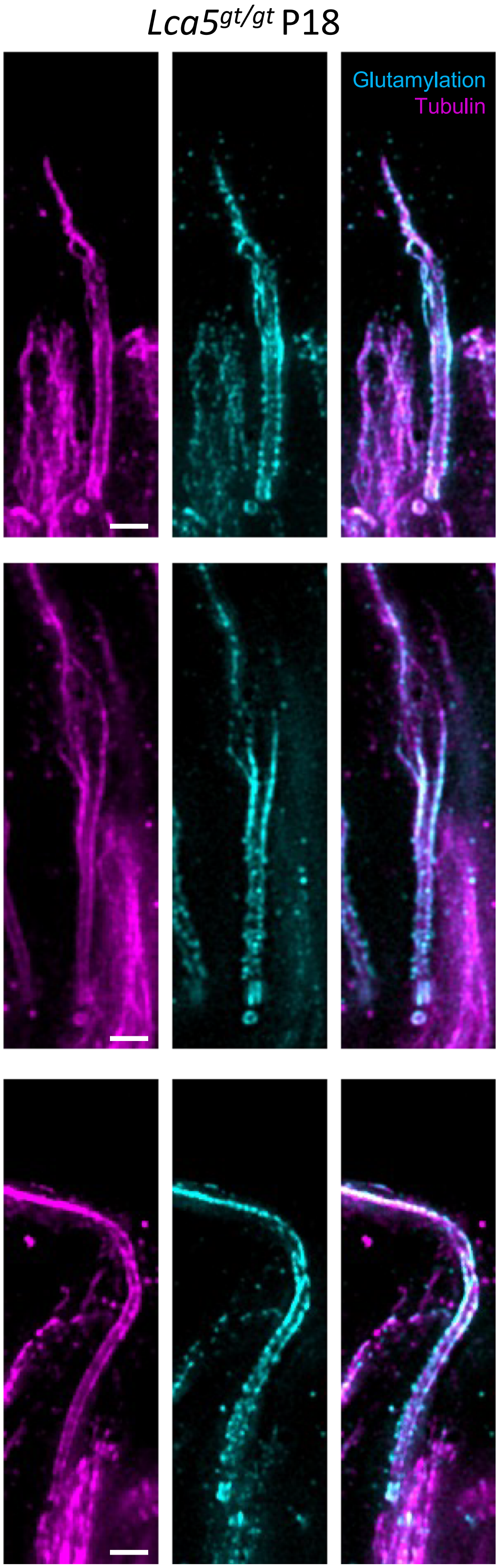

Figure EV5. Hyperglutamylation of the OS in *Lca5*$^{gt/gt}$ photoreceptor cells.

Expanded P18 *Lca5*$^{-/-}$ photoreceptor cells stained for glutamylation (GT335, cyan) and tubulin (magenta). Scale bar: 500 nm.

 © The Author(s)
